# ITS2 and 18S rRNA gene sequence-structure phylogeny of the Haptophyta (Haptista)

**DOI:** 10.1371/journal.pone.0344353

**Published:** 2026-03-19

**Authors:** Louisa Ridder, Bente Edvardsen, Matthias Wolf

**Affiliations:** 1 Department of Bioinformatics, Biocenter, University of Würzburg, Würzburg, Germany; 2 Department of Biosciences, University of Oslo (UIO), Oslo, Norway; CONICET: Consejo Nacional de Investigaciones Cientificas y Tecnicas, ARGENTINA

## Abstract

The phylogeny of haptophytes, a diverse and ecologically significant group of microalgae, remains incompletely resolved despite extensive molecular studies. In this study, we apply a sequence-structure phylogenetic framework to the Haptophyta, utilizing ribosomal RNA (rRNA) small subunit (SSU) gene (18S; nearly complete sequences) and internal transcribed spacer 2 (ITS2) datasets. By integrating secondary structure information during sequence alignment and tree inference, we aim to enhance phylogenetic resolution and clarify evolutionary relationships within this lineage. Our taxon sampling reduced over 40,000 available 18S sequences to 396 representatives, alongside a compilation of 224 ITS2 sequences. Comparative modeling and homology-based structure prediction revealed both conserved and variable features in 18S and ITS2 secondary structures, with notable deviations in certain taxa. Maximum likelihood (ML) subset phylogenies based on 18S sequence-structure data showed the greatest congruence with established taxonomy, such as the division between calcifying and non-calcifying lineages. In contrast, ITS2 data presented alignment challenges due to high sequence variability, length differences, and limited taxon representation. Incorporating secondary structure information improved alignment quality and reduced phylogenetic artifacts, though ITS2 remained unsuitable for resolving deep relationships among haptophytes. Instead, ITS2 proved more valuable for distinguishing closely related species. While bootstrap support values were similar between sequence-only and sequence-structure approaches, the latter suggested alternative phylogenetic placements that better aligned with previous studies (using multiple markers or also some partial 18S sequences); for *Hayaster perplexus* in particular, these placements also better matched morphological data. Our results underscore the critical impact of taxon sampling and methodological choices on phylogenetic outcomes. Despite these challenges, the 18S sequence-structure ML tree offers a reliable depiction of haptophyte phylogeny, even though some backbone relationships remain weakly supported. Overall, this study highlights both the benefits and limitations of integrating RNA secondary structure into molecular phylogenetics and advances our understanding of haptophyte evolution.

## Introduction

Understanding evolutionary relationships within and among eukaryotic lineages remains a central goal of molecular systematics. Traditionally, phylogenetic analyses have relied heavily on sequence data in addition to morphology, yet an increasing body of research demonstrates that incorporating secondary structure information from ribosomal RNA genes can substantially enhance phylogenetic resolution and support. Keller et al. [1] systematically evaluated the impact of integrating secondary structure into phylogenetic inference, showing that both the accuracy and robustness of resulting trees are markedly improved when this information is considered. These findings, consistent across various taxonomic levels and biological contexts, provide strong justification for the sequence-structure approach. Wolf et al. [2] further synthesized these methodological advances in a comprehensive review, highlighting the transformative potential of RNA sequence-structure phylogenetics for resolving complex evolutionary relationships.

Building on this foundation, several large-scale studies have successfully applied sequence-structure methods to diverse algal groups. For instance, Rackevei et al. [3] reconstructed the phylogeny of Euglenophyceae using 18S rDNA (ribosomal DNA) sequence and secondary structure data, resulting in improved tree robustness and higher support values compared to sequence-only analyses. Similarly, Berchtenbreiter et al. [4] employed both 18S and ITS2 sequence-structure data to resolve relationships within Phaeophyceae, demonstrating that structural information yields well-supported and congruent phylogenies. In green algae, Buchheim et al. [5] utilized 18S rRNA gene sequence-structure analyses to clarify relationships within Chlorophyceae, while Heeg and Wolf [6] applied a similar approach to *Chlorella* and related genera, successfully resolving complex lineages within the Chlorellaceae. Collectively, these studies underscore the value of integrating sequence and secondary structure data for robust phylogenetic inference across a broad spectrum of taxa.

Against this methodological background, we turn our focus to the Haptophyta, a clade of predominantly marine microalgae that play a crucial role in the global carbon cycle and marine food webs. Haptophytes are characterized by the unique haptonema, an organelle likely involved in feeding and surface attachment, and include the ecologically important coccolithophores, which produce calcareous plates known as coccoliths [7,8]. Despite their ecological significance, the phylogeny of haptophytes remains incompletely resolved. The eukaryotic supergroup Haptista, likely a sister group to the TSAR (telonemids, stramenopiles, alveolates, rhizaria) clade, is currently divided into two main phyla: Centrohelida and Haptophyta [9]. Within Haptophyta, three major groups (classes) have been identified: Pavlovophyceae, Prymnesiophyceae (also known as Coccolithophyceae), and Rappephyceae [10,11]. However, the phylogenetic position of Rappephyceae remains debated, with some studies suggesting it represents a deeply branching lineage and others placing it between Pavlovophyceae and Prymnesiophyceae. This ambiguity complicates the classification of Rappemonada versus a monophyletic Haptomonada clade [11,12]. Within Prymnesiophyceae, several orders have been recognized, including Coccolithales, Isochrysidales, Phaeocystales, Prymnesiales, Syracosphaerales, and Zygodiscales [13–17]. While molecular studies strongly support most of these orders [13,14], finer-scale relationships between and within these clades remain to be fully resolved.

In this study, we address these outstanding questions by applying a sequence-structure phylogenetic framework to the Haptophyta. Using ribosomal small subunit (18S) and internal transcribed spacer 2 (ITS2) data sets and integrating secondary structure information during alignment and tree inference, we aim to achieve improved resolution and a deeper understanding of evolutionary relationships within this diverse and ecologically important lineage.

## Materials and methods

The generation of 18S and ITS2 data sets (taxon sampling) is described in supplementary materials (S1 Table). Detailed methods for alignment and tree reconstruction are outlined in Rackevei et al. and Berchtenbreiter et al. [3,4]. Briefly, in contrast to sequence-only analyses, we used 12x12 scoring matrices for automatic RNA sequence-structure alignments and substitution models for phylogenetic tree reconstructions. Our encoding combines RNA sequence and structural information into 12 letters derived from standard amino acid abbreviations [2]. Each letter represents a nucleotide paired with its structural context (unpaired, paired left, or paired right in the secondary structure). This encoding makes RNA data compatible with existing bioinformatics tools designed for amino acids.

Instead of the usual 20x20 amino acid scoring matrix and substitution model, we used specially adapted 12x12 versions that define similarity scores and model parameters between encoded letters. This approach allows efficient use of bioinformatics tools with minimal modifications while simultaneously considering RNA sequence and structural information. Consequently, analyses using Neighbor-Joining (NJ) [18], Profile-Neighbor-Joining (PNJ) [19], Maximum Parsimony (MP) [20], and Maximum Likelihood (ML) [21] benefit from an enhanced pipeline for RNA sequence-structure phylogenetics.

We used the following tools: GenBank [22], Align [23], RNAcentral [24], the ITS2 database [25,26], RNAstructure [27], ClustalX/ClustalW [28], 4SALE [29,30], ProfDistS [31,32], PAUP* [33], phangorn [34], and MEGA [35]. The latter four tools were used in combination with a bootstrap analysis [36]. Results from sequence-structure analyses are contrasted with those obtained via classic sequence-only approaches. Any deviations from default parameter settings are detailed in figure legends. All alignments are available as supplementary material (S1–S8 Files).

## Results

### 18S rRNA gene

**Fig 1:**
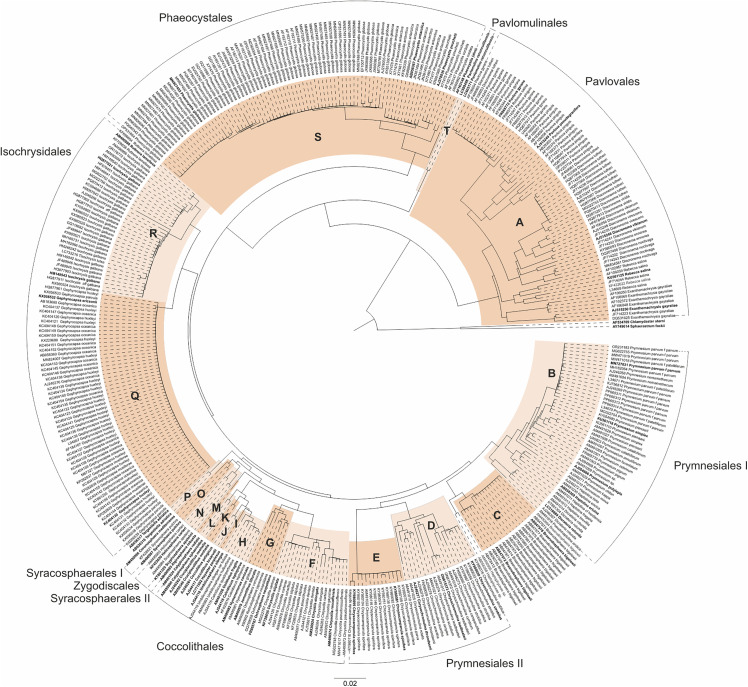
Neighbor-Joining (NJ) tree based on 18S rDNA sequence-structure data for 396 haptophytes and two centrohelids (outgroup). The sequence-structure alignment was generated using 4SALE. Species names are accompanied by their respective GenBank accession numbers. Clades selected for subsequent profile Neighbor-Joining (PNJ) analyses are alternately colored, and sequences used in later subset analyses are highlighted in bold. Families and orders within Prymnesiophyceae are labeled **A**–**T** as follows: **A**: Pavlovaceae, **B**: Prymnesiaceae I, **C**: Braarudosphaeraceae, **D**: Chrysochromulinaceae, **E**:Prymnesiaceae II, **F**:Chrysotilaceae, **G**:Hymenomonadaceae, **H**: Calcidiscaceae I, **I**: *Hayaster perplexus* (Calcidiscaceae **II)**, **J**: Coccolithaceae I, **K**: Coccolithaceae II, **L**: Calyptrosphaeraceae, **M**: Rhabdosphaeraceae, **N**: Zygodiscales (including Pontosphaeraceae & Helicosphaeraceae), **O**: Syracosphaeraceae, **P**: *Tergestiella adriatica* (Watznaueriaceae), **Q**: Noelaerhabdaceae, **R**: Isochrysidaceae, **S**: Phaeocystaceae, **T**: Pavlomulinaceae. According to [37–42] some strains have been renamed here in contrast to NCBI (for outdated names see S1 Table). Orders are additionally annotated around the tree. The scale bar and branch lengths represent evolutionary distances.

Based on our taxon sampling, we reduced the approximately 40,000 Haptophyta 18S sequences currently listed in GenBank to 396 representative sequences for further analysis (see S1 Table and Fig 1).

All 18S secondary structures were predicted by comparative modeling, using a reference structure from the RNAcentral database [24] (*Prymnesium polylepis*, GenBank accession: AJ004866) as a template. Each predicted 18S structure shared at least 80% identical (homologous) base pairings with the template.

The sequence-structure overview tree (Fig 1) encompassed eight orders, of which all but two-Prymnesiales and Syracosphaerales-were monophyletic. The order Prymnesiales appeared paraphyletic as the taxon *Braarudosphaera bigelowii* fell in the clade Prymnesiales but presently belongs to the order Braarudosphaerales. Syracosphaerales appeared paraphyletic, showing sister relationships with Zygodiscales (Syracosphaerales I) and Coccolithales (*Algirosphaera robusta*). Fifteen of the seventeen families formed monophyletic groups, whereas Prymnesiaceae and Coccolithaceae appeared paraphyletic. Two sequences (wrongly classified as *Haptolina brevifila* (strain MBIC19518 and strain Kawachi) in NCBI), here labelled as Haptophyceae sp. (Genbank accession AM490995, AB058358), could not be assigned to any established clade. Prymnesiaceae were divided into *Chrysocampanula spinifera* organisms and the remaining prymnesiacean species by the Braarudosphaerales and Chrysochromulinaceae falling into the clade. The coccolithacean paraphyly resulted from two *Coccolithus* organisms sistering the Calcidiscaceae whereas two *Cruciplacolithus* organisms were sister to the clade resulting from that. Pavlovales, representing the Pavlovophyceae, formed the sister group to all other haptophytes. Subsequently, the order Pavlomulinales within the class Rappephyceae were recovered as the sister group to Prymnesiophyceae. Within Prymnesiophyceae, Phaeocystales occupied the basal position. Isochrysidales was sister to the rest of Prymnesiophyceae. Braarudosphaeraceae and Prymnesiales I corresponding to the family Prymnesiaceae (including *Prymnesium*, *Dicrateria*, *Haptolina*, and *Pseudohaptolina*) were sister to Prymnesiales II (including *Chrysochromulina* in the family Chrysochromulinaceae and *Chrysocampanula spinifera*). The Prymnesiales/Braarudosphaeraceae clade was in turn sister to a clade with *Tergestiella adriatica* (taxonomically placed in Coccolithales) at its base. Within this clade, Syracosphaerales I (including the two syracosphaeracean genera *Syracosphaera* and *Coronosphaera*) grouped with Zygodiscales, forming the sister group to a clade consisting of Coccolithales and Syracosphaerales II (including *Algirosphaera robusta)*.

The sequence-only overview tree (S1 Fig) recovered nearly the same mono- and paraphyletic clades as the sequence-structure tree. However, notable differences emerged within the Prymnesiophyceae. In this group, the Prymnesiales/Braarudosphaerales clade appeared as the sister group to Isochrysidales, and together they formed a sister lineage to a clade comprising Coccolithales, Zygodiscales, Syracosphaerales, and *Tergestiella adriatica*. Within Coccolithales, Calcidiscaceae were not recovered as monophyletic: Calcidiscaceae I grouped as sister to Coccolithaceae, while *Hayaster perplexus* (Calcidiscaceae II) was positioned at the base of this clade.

**Fig 2:**
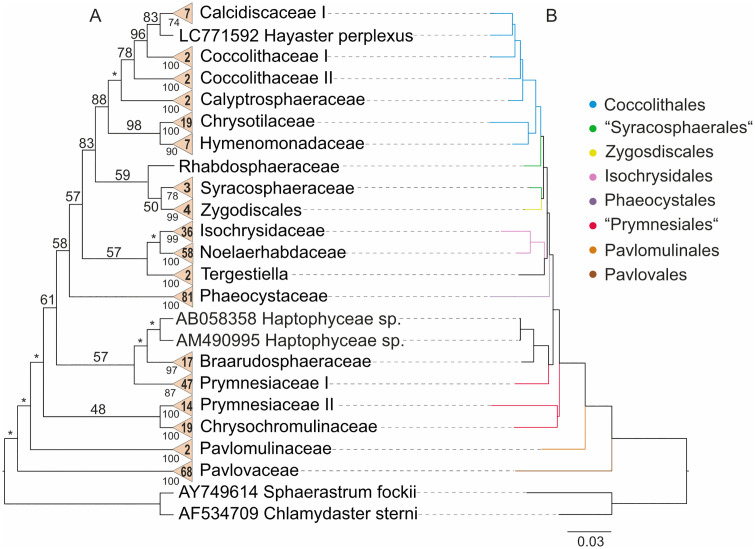
Profile Neighbor-Joining (PNJ) tree based on 18S rDNA sequence-structure profiles for 396 organisms. The tree was generated using ProfDistS, and the sequence-structure alignment was generated using 4SALE. Species names are accompanied by their respective GenBank accession numbers. Two strains (Genbank accession AM490995, AB058358) were wrongly classified in NCBI and here renamed to Haptophyceae sp. (for outdated names see S1 Table). They could not be assigned to any clade [37,38]. **A:** The cladogram on the left shows a three-times iterated PNJ tree. Bootstrap values from 100 pseudo-replicates are displayed at internal nodes. During each iteration, super-profiles were generated using existing profiles and bootstrap values >75. Numbers within the triangles indicate the number of sequences in manually defined profiles. Profile bootstrap values were derived and transferred from bootstrap testing (100 pseudo-replicates) performed on the overview NJ tree (Fig 1). **B:** The phylogram on the right depicts the original PNJ tree without further iterations. Both trees are rooted with *Chlamydaster sterni* (GenBank accession AF534709) and *Sphaerastrum fockii* (GenBank accession AY749614). Branch lengths and the scale bar represent evolutionary distances. Colours indicate the corresponding haptophyte orders as follows: **blue**: Coccolithales, **green**: Syracosphaerales, **yellow**: Zygodiscales, **pink**: Isochrysidales, **violet**: Phaeocystales, **red**: Prymnesiales, **orange**: Pavlomulinales, **brown**: Pavlovales.

All major clades in the PNJ sequence-structure trees (Fig 2) were highly supported, with bootstrap values exceeding 73. Consistent with the overview trees, both Prymnesiaceae and Coccolithaceae, as well as Prymnesiales and Syracosphaerales, were paraphyletic. The base of the tree was formed by Pavlovales, followed by Pavlomulinales, both of which represented well-supported, distinct lineages (classes). Prymnesiales II (with low support), including *Chrysochromulina* (Chrysochromulinaceae) and *Chrysocampanula* (Prymnesiaceae II), were sister to a moderately supported clade comprising all remaining haptophytes. A moderately supported Braarudosphaeraceae/Prymnesiales I clade was sister to Phaeocystales plus a clade containing Coccolithales, Isochrysidales, Syracosphaerales, *Tergestiella*, and Zygodiscales; the latter clade, however, received only moderate support. Isochrysidales were fully supported and formed a clade together with *Tergestiella* (BS (bootstrap support)=57), both of which were sister to the remaining taxa (BS = 83). Coccolithales were well supported (BS = 88) and formed a sister group to a moderately supported clade comprising Syracosphaerales and Zygodiscales. Within Coccolithales, *Hayaster perplexus* formed a monophyletic clade with the other calcidiscacean taxa (BS = 83). Coccolithaceae I (*Coccolithus*) and Coccolithaceae II (*Cruciplacolithus*) each represented distinct lineages. In the Syracosphaerales/Zygodiscales clade, family Rhabdosphaeraceae (Syracosphaerales II) occupied a basal position, with Syracosphaerales I (including *Syracosphaera* and *Coronosphaera*) and Zygodiscales forming the respective sister groups (moderately supported).

In contrast to the sequence-structure PNJ tree, the monophyly of Calcidiscaceae was not supported in the sequence-only PNJ tree (S2 Fig). Here, Calcidiscaceae I was again recovered as sister to Coccolithaceae I, while *Hayaster perplexus* (Calcidiscaceae II) was positioned at the base of this clade.

The average bootstrap backbone support for the sequence-structure PNJ tree was slightly higher (79.7) (Fig 2) than for the sequence-only PNJ tree (77.8) (S2 Fig).

The latter analysis (sequence-only PNJ) recovered almost the same paraphyletic orders and families as observed in the sequence-structure PNJ trees (Fig 2). All profiles received bootstrap support values exceeding 64. Due to short evolutionary distances and low bootstrap support, the original and iterated trees displayed differing topologies. Within the strongly supported Prymnesiophyceae, Phaeocystales occupied a basal position as sister to all other prymnesiophycean haptophytes, albeit with moderate support. Prymnesiales, in contrast to the sequence-structure tree (Fig 2), were recovered as monophyletic (moderate support) and formed a sister group to Braarudosphaeraceae, though with low support. Together, these two clades were sister to a well-supported clade comprising all remaining taxa. Coccolithales were robustly supported and were sister to a weakly supported clade containing Isochrysidales (highly supported), Syracosphaerales, *Tergestiella*, and Zygodiscales. Within this group, a poorly supported Isochrysidales/*Tergestiella* clade was sister to a highly supported Syracosphaerales/Zygodiscales clade (moderate support). In the latter, Syracosphaerales I (embracing family Syracosphaeraceae) formed the basal lineage, sistering a clade consisting of Zygodiscales and Syracosphaerales II (family Rhabdosphaeraceae) (BS = 55).

**Fig 3:**
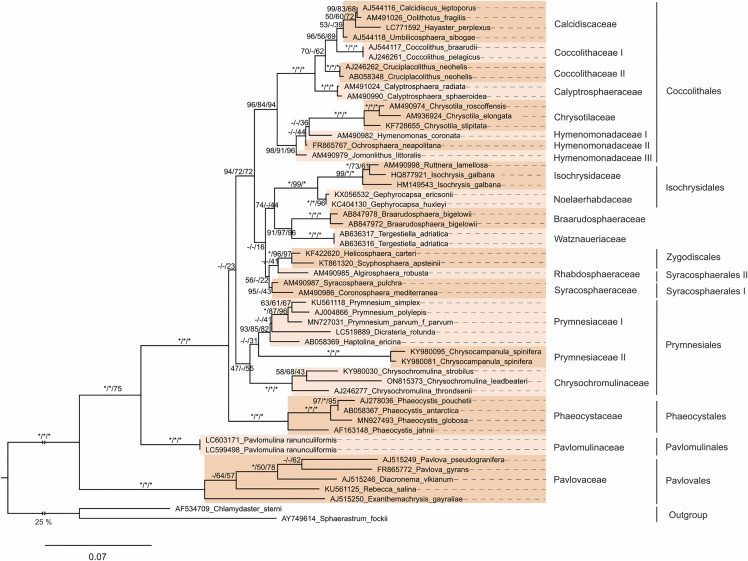
Maximum-likelihood (ML) tree based on 18S rDNA sequence-structure data for a manually chosen subset of 53 organisms representing the profiles used in the PNJ analysis. The tree was constructed in R using the *phangorn* package. Model parameters (GTR + I + G) were estimated from the data. The sequence-structure alignment was generated using 4SALE. According to [39–42] some species were renamed in this study in contrast to NCBI taxonomy (cf. S1 Table). Alternating colors highlight the PNJ profiles, and family and order names are displayed on the right side of the tree. Non-monophyletic orders are indicated either with quotation marks or by Roman numerals. The branch length of the outgroup (*Sphaerastrum fockii* and *Chlamydaster sterni*) is trimmed to 25%. Branch lengths and the scale bar represent evolutionary distances. Bootstrap values are shown for three methods—neighbor-joining (NJ, obtained in ProfDistS), maximum-parsimony (MP, obtained in PAUP), and maximum-likelihood (ML)—in the order NJ/MP/ML. A bootstrap value of 100 is marked with an asterisk (*), while a dash (-) indicates differing tree topologies across methods.

Within the sequence-structure subset tree (NJ, MP, and ML), all orders except Syracosphaerales were recovered as monophyletic (Fig 3). At the base of the tree, Pavlovales and Pavlomulinales were successively and robustly supported. Pavlomulinales formed the sister group to the fully supported Prymnesiophyceae. Within Prymnesiophyceae, Phaeocystales (fully supported) occupied the basal position of the clade, albeit with low support and only in the ML analysis. Prymnesiales received low to moderate support (47/-/56); within this order, Prymnesiaceae were monophyletic (BS = -/-/31) and formed a sister group to Chrysochromulinaceae. Adjacent to Prymnesiales, a moderately supported clade (94/72/72) comprised the remaining prymnesiophycean taxa. The basal group within this class were the highly supported Coccolithales. However, within Coccolithales, only three of the five families were monophyletic. Coccolithaceae and Hymenomonadaceae were not recovered as monophyletic. As seen before, *Coccolithus* organisms were sister to the calcidiscacean family, whereas *Cruciplacolithus neohelis* stood at the base of this group. Into the Hymenomonadaceae clade fell the chrysotilacean family. The clade of the remaining haptophytes had only low support (-/-/16). A highly supported clade consisting of Braarudosphaeraceae and *Tergestiella* was sister to the fully supported Isochrysidales (100/99/100), with this relationship receiving low to moderate support (74/-/44). Together, these two clades were sister to a weakly supported clade (56/-/22), in which Syracosphaerales II (*Algirosphaera robusta*) grouped with Zygodiscales (low support) and was set apart from Syracosphaerales I (including *Syracosphaera* and *Coronosphaera*).

In the sequence-only subset trees (S3 Fig), six out of eight orders were recovered as monophyletic, while Syracosphaerales and Prymnesiales appeared paraphyletic. Compared to the sequence-structure subset tree (Fig 3), the topologies within the fully supported coccolithophycean clade differed notably. At the base, the fully supported Prymnesiales II (including Chrysochromulinaceae) formed the sister group to a weakly supported clade comprising the remaining haptophytes. Prymnesiaceae were monophyletic, representing Prymnesiales I (with low support), and were sister to another weakly supported clade containing Braarudosphaeraceae, Coccolithales, Isochrysidales, Phaeocystales, Syracosphaerales, *Tergestiella*, and Zygodiscales. This large clade was further divided into two sister groups. The first, which received almost no support, consisted of the highly supported Isochrysidales and the fully supported Phaeocystales. The second clade, with only low support, comprised a highly supported clade of *Tergestiella* and Braarudosphaeraceae, followed by highly supported Coccolithales (in which Coccolithaceae and Calcidiscaceae were not monophyletic) as sister to a moderately supported Syracosphaerales/Zygodiscales clade (BS = 96/-/49). Within this latter clade, Syracosphaerales II was sister to a group comprising Zygodiscales and Syracosphaerales I (including *Syracosphaera* and *Coronosphaera*) (BS = -/-/41).

### ITS2

**Fig 4:**
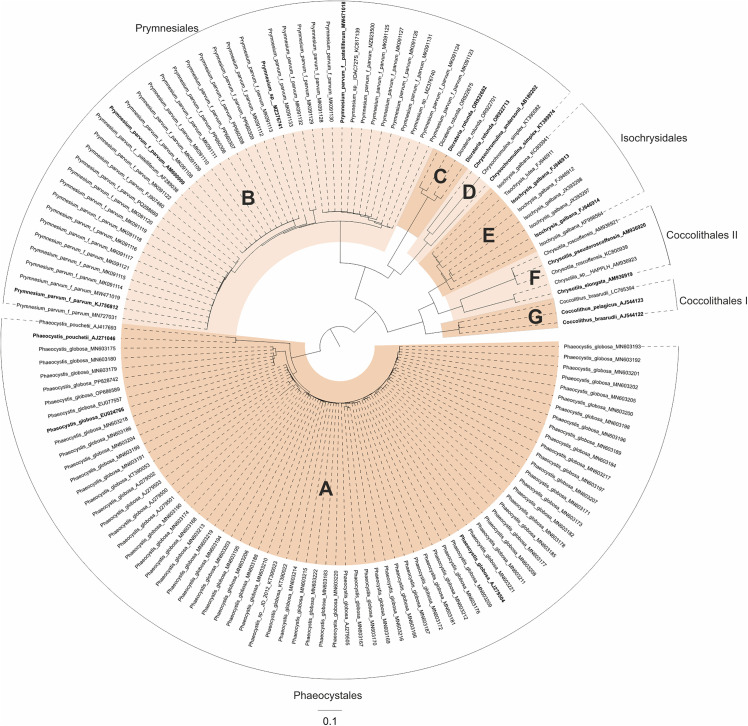
Neighbor-Joining (NJ) tree based on ITS2 rDNA sequence-structure data for 137 haptophytes. The sequence-structure alignment was generated using 4SALE. Species names are accompanied by their respective GenBank accession numbers. As the outgroup, Phaeocystales was chosen. Some species names were exchanged here in contrast to NCBI taxonomy according to [39,41,42] (for outdated names see S1 Table). Clades selected for subsequent profile Neighbor-Joining (PNJ) analyses are alternately colored, and sequences used in later subset analyses are highlighted in bold. Families and orders within Prymnesiophyceae are labeled **A**–**G** as follows: **A**: *Phaeocystis*, **B**: *Prymnesium*, **C**: *Dicrateria*, **D**: *Chrysochromulina I*, **E**: *Isochrysis*, **F**: *Chrysotila*, **G**: *Coccolithus*. Orders are additionally annotated around the tree. The scale bar and branch lengths represent evolutionary distances.

**Fig 5:**
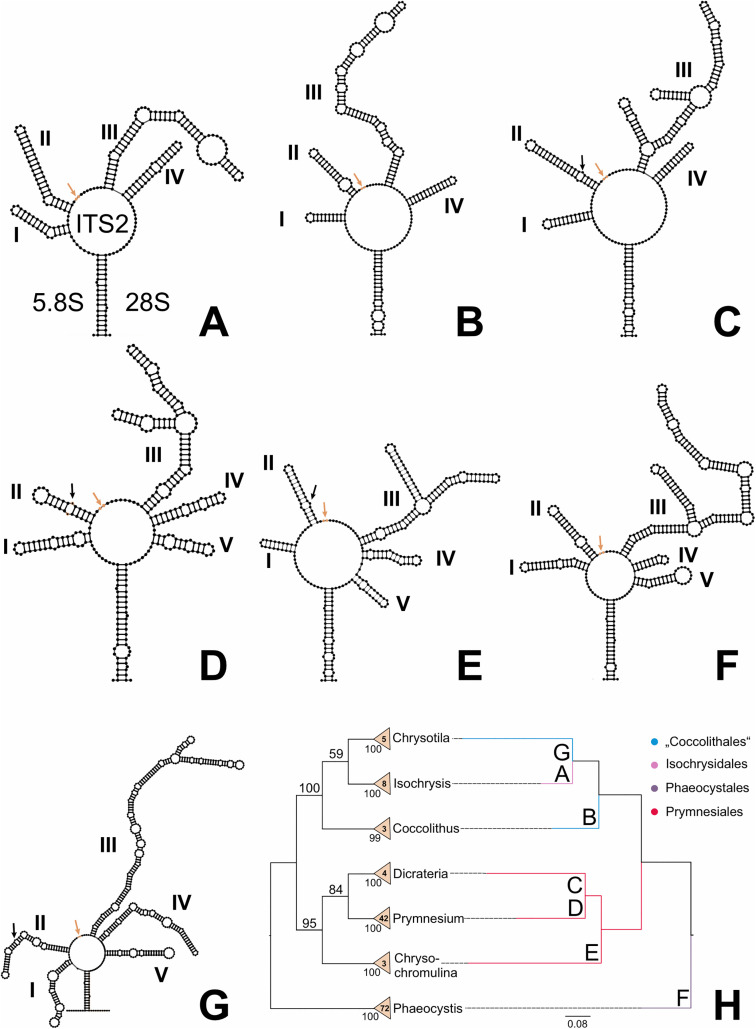
ITS2 secondary structure overview and sequence-structure Profile Neighbour-Joining tree. From **A** to **G,** secondary structures for the ITS2 rDNA of seven haptophyte organisms are depicted. They were visualized in VARNA and are labelled as follows: **A**: *Isochrysis galbana* (GenBank accession: FJ946913), **B**: *Coccolithus braarudii* (GenBank accession: AJ44122), **C**: *Dicrateria rotunda* (GenBank accession: OR922682), **D**: *Prymnesium parvum f. parvum* (GenBank accession: AM690999), **E**: *Chrysochromulina simplex* (GenBank accession: KT380974), **F**: *Phaeocystis globosa* (GenBank accession: AJ279504), **G**: *Chrysotila pseudoroscoffensis* (GenBank accession: AM936920). The helices are given roman numerals. A red arrow indicates an A-rich region between the second and third helix, a black arrow a U-U mismatch at the second helix; both typical traits of the ITS2. **H** shows a profile Neighbour-Joining (PNJ) tree based on ITS2 rDNA sequence-structure profiles for 137 organisms. The tree was generated using ProfDistS, and the sequence-structure alignment was generated using 4SALE. The cladogram on the left shows a three-times iterated PNJ tree. Bootstrap values from 100 pseudo-replicates are displayed at internal nodes. During each iteration, super-profiles were generated using existing profiles and bootstrap values >75. Numbers within the triangles indicate the number of sequences in manually defined profiles. Profile bootstrap values were derived and transferred from bootstrap testing (100 pseudo-replicates) performed on the overview NJ tree (Fig 4). The phylogram on the right depicts the original PNJ tree without further iterations. Both trees are rooted with Phaeocystales. Branch lengths and the scale bar represent evolutionary distances. Colours indicate the corresponding haptophyte orders as follows: **blue**: Coccolithales, **pink**: Isochrysidales, **violet**: Phaeocystales, **red**: Prymnesiales.

For the ITS2 analysis, a dataset of 224 haptophyte organisms was compiled (cf. Fig 4). Sixty-two sequences were excluded due to their short length, most of which belonged to the genera *Phaeocystis* or *Prymnesium*. Despite these exclusions, both genera remained well represented. To obtain ITS2 structural information for each sequence, homology modeling was performed using seven different templates obtained by energy minimization and constraint folding (Fig 5). An additional 24 organisms, whose structural homology was below 65%, were also removed. The final dataset consisted of 137 haptophyte organisms, all belonging to the class Prymnesiophyceae.

All ITS2 secondary structures exhibited a characteristic proximal stem containing two unpaired nucleotides [26,43]. Each structure also displayed one of the three typical ITS2 sequence motifs: an A-rich region located between helices II and III. A second common motif, a U-U mismatch in helix II, was identified in *Dicrateria*, *Prymnesium*, *Chrysochromulina*, and *Chrysotila*. The third well-known ITS2 motif, a UGGU sequence at the 5’ apex of helix III, could not be clearly identified in this group of organisms. The canonical eukaryotic ITS2 secondary structure-comprising four helices, with the third being the longest-was observed in *Isochrysis*, *Coccolithus*, and *Dicrateria*. In contrast, *Prymnesium*, *Chrysochromulina*, *Phaeocystis*, and *Chrysotila* exhibited a fifth helix, a feature also reported in certain other eukaryotes. These four taxa also displayed additional small helical side arms, representing deviations from the typical ITS2 structure. *Dicrateria*, in particular, exhibited two such side arms on the third helix. Notably, the ITS2 sequence of *Chrysotila* was exceptionally long, comprising 696 nucleotides—approximately twice the length observed in other Haptophyta (Figs 5 and S4).

The sequence-structure overview tree (Fig 4) revealed four haptophyte orders, three of which were monophyletic. Only the order Coccolithales appeared paraphyletic. All families were monophyletic. In this analysis, following the 18S trees, Phaeocystales served as the outgroup. At the base of the tree, Prymnesiales formed the sister group to a larger clade that included both Isochrysidales and Coccolithales. Within this clade, Isochrysidales together with Coccolithales II (including *Chrysotilaceae*) were sister to Coccolithales I (including *Coccolithus*).

In the sequence-only overview tree (S5 Fig), two of the four orders were monophyletic, while Coccolithales and Prymnesiales appeared not monophyletic. The genus *Chrysochromulina* was also divided, resulting in paraphyly. Using *Phaeocystis* as the outgroup, the tree was resolved into six groups in the following ascending order: Prymnesiales III (including *Chrysochromulina andersonii*), Isochrysidales, Prymnesiales II (including *Chrysochromulina simplex*), Coccolithales II (including *Chrysotila*), *Coccolithales* I (including *Coccolithus*), and Prymnesiales I (including *Prymnesium* and *Dicrateria*).

The sequence-structure PNJ tree (Fig 5H) showed three monophyletic orders, while Coccolithales remained paraphyletic. All profiles received full or high support. In this tree, Phaeocystales were used as the outgroup and were sister to a clade in which the well-supported Prymnesiales formed the basal group. This was followed by a fully supported clade comprising both Coccolithales and Isochrysidales. Within this group, Coccolithales I (including *Coccolithus*) was sister to a moderately supported group consisting of Coccolithales II (including *Chrysotila*) together with *Isochrysis*.

The original and iterated sequence-only PNJ trees (S6 Fig) differed at nodes with low support. Due to artifacts- mainly caused by the considerable length differences among ITS2 sequences, which disrupted the sequence-only alignment-profile bootstrap values could not be reliably determined. In the original PNJ tree, Isochrysidales and Phaeocystales were monophyletic, while Coccolithales and Prymnesiales were not monophyletic. Of the seven genera, six were monophyletic, while *Chrysochromulina* was paraphyletic. After *Phaeocystis* as outgroup, the tree split into two weakly supported clades: one comprised *Chrysochromulina* I as sister to the weakly supported *Chrysochromulina* II and *Chrysotila*; in the other, *Isochrysis* formed the basal group, sister to a fully supported clade in which *Coccolithus* was sister to a strongly supported group of *Prymnesium* and *Dicrateria*.

**Fig 6:**
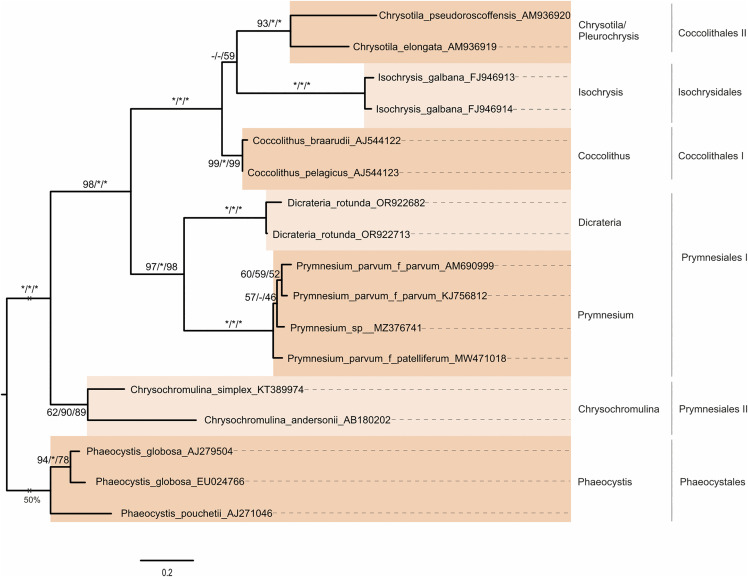
Maximum-likelihood (ML) tree based on ITS2 rDNA sequence-structure data for a manually chosen subset of 17 organisms representing the profiles used in the PNJ analysis. The tree was constructed in R using the *phangorn* package. Model parameters (GTR + I + G) were estimated from the data. The sequence-structure alignment was generated using 4SALE. According to [39,41] species names here differ from names in the NCBI database (cf. S1 Table). Alternating colors highlight the PNJ profiles. Genera and order names are displayed on the right side of the tree. Non-monophyletic orders are indicated by Roman numerals. The branch length of the outgroup Phaeocystales is trimmed to 50%. Branch lengths and the scale bar represent evolutionary distances. Bootstrap values are shown for three methods—neighbor-joining (NJ, obtained in ProfDistS using the Q-ITS2 model), maximum-parsimony (MP, obtained in PAUP), and maximum-likelihood (ML)—in the order NJ/MP/ML. A bootstrap value of 100 is marked with an asterisk (*), while a dash (-) indicates differing tree topologies across methods.

In the sequence-structure subset trees (Fig 6), all genera were monophyletic. Of the four orders, two were monophyletic, while Coccolithales and Prymnesiales were paraphyletic. The outgroup Phaeocystales was fully supported. Prymnesiales II (including *Chrysochromulina*) formed a strongly supported, distinct lineage that was sister to a highly supported clade containing Coccolithales, Isochrysidales, and Prymnesiales I. At the base of this latter clade were Prymnesiales I, including the genera *Prymnesium* and *Dicrateria* (highly supported). The remaining taxa formed a fully supported clade. Within this group, Coccolithales I (including *Coccolithus*) was strongly supported as sister (BS = -/-/59) to a group comprising fully supported Isochrysidales and Coccolithales II (including *Chrysotila*).

The sequence-only subset trees (S7 Fig) included four orders, with Prymnesiales and Coccolithales appearing non-monophyletic. All genera were monophyletic and received high or full support. After *Phaeocystis* (Phaeocystales) as outgroup, *Isochrysis* (Isochrysidales) formed the basal group, sister to the remaining haptophytes (highly supported). *Chrysotila* (Coccolithales II) was sister to a strongly supported group with *Chrysochromulina* (Prymnesiales III) at its base. A clade containing *Dicrateria*, *Coccolithus*, and *Prymnesium* had low support; within this, the sister relationship between *Coccolithus* and *Prymnesium* was moderately supported by only the maximum likelihood analysis (BS = -/-/53).

To get a summary of the most important findings from comparing all 18S and ITS2 trees see S2 Table.

## Discussion

Current molecular phylogenetic frameworks of the Haptophyta reveal a well-defined hierarchical structure within the group. The class Pavlovophyceae are consistently recovered as the basal lineage, forming the sister group to all other haptophytes. Among the remaining Haptophyta, mainly Prymnesiophyceae, a major split distinctly separates the non-calcifying orders-most notably the Phaeocystales and Prymnesiales-from the mostly calcifying calcihaptophycean lineages, which include Coccolithales, Isochrysidales (of which Isochrysidaceae are not calcifying), Syracosphaerales, and Zygodiscales as well as Braarudosphaeraceae. This division is robustly supported by multiple molecular markers, such as 18S rDNA and *rbcL* gene sequences, and is further corroborated by ultrastructural and ecological characteristics (e.g., [7,37,44]). Within the calcifying orders, distinct clades are resolved, with Coccolithales and Isochrysidales exhibiting a particularly close relationship, while Syracosphaerales and Zygodiscales represent additional, well-supported lineages based on coccolith morphology [45]. Furthermore, numerous fossil orders, including Discoasterales and Eiffellithales, are placed within the calcifying clade, underscoring the evolutionary depth and complexity of coccolithophore diversification.

Direct comparison of our results with those reported in the literature remains challenging, primarily due to differences in taxon sampling strategies. Many previous studies rely on nearly complete or partial 18S rRNA sequences and/or combine fragments of the 18S gene with other molecular markers. In contrast, our analyses are based exclusively on nearly complete 18S sequences, which allowed us to incorporate secondary structure information. The variety of methodological approaches employed in earlier studies further complicates direct comparisons. Nonetheless, at least concerning the big picture, our findings align with the current consensus in the literature.

Notably, our maximum likelihood (ML) tree constructed using both sequence and secondary structure information of 18S rRNA demonstrated the greatest congruence with established groupings (e.g., calcifying versus non-calcifying taxa). Conversely, the ML tree based solely on sequence data appeared more susceptible to artifacts, particularly in the placement of Phaeocystales and did not fully reflect the known large clade distribution of haptophytes.

Both profile neighbor-joining (PNJ) trees-whether based solely on sequence data or on combined sequence-structure information-corresponded with the established “big picture” distribution of all haptophyte taxa, with the exception that, in the sequence-structure PNJ tree, the Phaeocystales again occupied a divergent position within the non-calcifying group within class Prymnesiophyceae, deviating from the consensus reported in the literature. Instead of standing at the base of the Prymnesiophyceae, Phaeocystales was found further within the group.

The two overview trees recovered the major clades within the Haptophyta as expected; however, relationships among these clades remain largely unresolved at shallow evolutionary depths. Interestingly, the topology of the sequence-only overview tree more closely resembled the consensus topology reported in the literature for Isochrysidales and Prymnesiales with Braarudosphaerales changed position in the sequence-structure overview tree.

In summary, both PNJ analyses (sequence-only or sequence-structure) and maximum likelihood analyses incorporating sequence-structure information from nearly complete 18S sequences are capable of recovering and largely supporting the consensus phylogeny (at least concerning the big picture) described in the literature.

For analyses based solely on sequence data, broader taxon sampling appears to mitigate potential artifacts.

However, besides this big picture, subgroups are now discussed below in more detail. As mentioned above, the classification of haptophytes based on their ability to calcify typically distinguishes two major groups. The first comprises the subclass Calcihaptophycidae, which includes the Coccolithales, Isochrysidales, Syracosphaerales, Zygodiscales, as well as the families Braarudosphaeraceae and Watznaueriaceae. Within this clade, all haptophytes are calcifying except for the family Isochrysidaceae within the Isochrysidales. The second group encompasses the remaining non-calcifying haptophytes, such as the Prymnesiales, Phaeocystales, Pavlovales, and Pavlomulinales. In the context of this division, the sequence-only overview phylogenetic tree showed a clearer separation between these groups than the sequence-structure tree, where no distinct relationships between calcihaptophycean and non-calcifying lineages could be discerned. Notably, the apparent division based on calcification observed in the sequence-only overview tree, as well as in both PNJ trees, was disrupted by the calcifying Braarudosphaeraceae, which formed a clade with the non-calcifying Prymnesiales. This relationship shifted in the Maximum Likelihood (ML) trees, where Braarudosphaeraceae was placed as sister to *Tergestiella*, thereby aligning with the calcihaptophycean clade. In the sequence-structure ML tree, the calcihaptophycean group was even recovered as monophyletic, whereas it appeared paraphyletic in the corresponding sequence-only tree due to Phaeocystales being sister to Isochrysidales. Overall, the sequence-structure ML tree was the only reconstruction to display a clear partitioning between Calcihaptophyceae and non-calcifying haptophytes, consistent with findings from previous studies [13]. Braarudosphaeraceae, only represented by the species *Braarudosphaera bigelowii*, including the life cycle stage originally described as *Chrysochromulina parkae* (cf. S1 Table), has a unique type of calcified scales differing from all other haptophytes called pentaliths [46]. The “*Chrysochromulina parkae”* life cycle stage is non-calcifying and resembles in morphology members of Prymnesiales. The phylogeny of this taxon appears unresolved [46].

### Pavlovales

The phylogenetic position and composition of the Pavlovales, and thus the pavlovophycean class, are already well established [10]. In all trees generated in this study, Pavlovales consistently formed a well-supported basal group to all other haptophytes, in agreement with previous findings [10,14,44].

### Pavlomulinales

The two sequenced strains included in the class Rappephyceae, order Pavlomulinales, Haptophyceae sp. NIES-3900 and Haptophyta sp. RCC 3430, have been classified as *Pavlomulina ranunculiformis* that belong to the rappemonads first described by Kawachi et al. [11]. In both the sequence-structure and sequence-only analyses, these taxa consistently formed a fully supported clade positioned between the Pavlovophyceae and Prymnesiophyceae. Despite the relatively recent discovery of this group, similar phylogenetic placement and support for the clade have been reported in other studies [11,14].

### Phaeocystales

Previous studies have suggested that the Phaeocystales occupy a basal position within the Prymnesiophyceae [13–15,44], with this placement receiving moderate [14] to high support [13,44]. In our analyses, the Phaeocystales clade was always strongly supported; however, its exact phylogenetic position and the level of support varied depending on the tree. In most trees generated in this study, Phaeocystales were also recovered as the basal group of the Prymnesiophyceae. Exceptions were observed in the sequence-structure PNJ tree and the sequence-only subset tree. In the iterated sequence-only PNJ tree, Phaeocystales occupied the same position as reported in the literature, as sister to a moderately supported clade of coccolithophycean haptophytes. However, due to the region of small evolutionary distances, their position differed in the original tree. The sequence-structure PNJ trees mirrored the position found in the sequence-only original PNJ tree, where Phaeocystales followed a moderately supported Prymnesiaceae I/Braarudosphaeraceae clade and were sister to a moderately supported clade comprising Coccolithales, Isochrysidales, Syracosphaerales, *Tergestiella*, and Zygodiscales. In the sequence-only subset tree, Phaeocystales formed a nearly unsupported clade with Isochrysidales, located in a region characterized by generally low bootstrap values. Although the basal position of Phaeocystales to Prymnesiophyceae was also recovered in the sequence-structure subset tree, this placement was not well supported but nonetheless corresponds to the position indicated in the literature.

### Prymnesiales

Most studies have recovered Prymnesiales as a monophyletic group, typically with moderate support [37,44,47,48]. This lineage generally branches after Phaeocystales and is sister to the remaining coccolithophycean haptophytes [7,14,44]. Within Prymnesiales, the Chrysochromulinaceae are often positioned basally to a Prymnesiaceae clade, supported by high statistical values [13,37]. An exception is reported by Hagino et al. [49], where Prymnesiales were not monophyletic and occupied a different position; however, the key nodes in that analysis lacked bootstrap support. In our own phylogenetic reconstructions, the position and composition of Prymnesiales varied. In both overview trees, Braarudosphaeraceae were nested within Prymnesiales. In the sequence-only overview tree, the clade matched the position found in [7,14,44], while in the sequence-structure overview tree, the Prymnesiales/Braarudosphaeraceae clade branched after Isochrysidales. Prymnesiales were first recovered as monophyletic (with moderate support) in the sequence-only PNJ tree, forming a weakly supported clade with Braarudosphaeraceae, which also branched after Phaeocystales. In contrast, the sequence-structure PNJ tree did not support Prymnesiales as monophyletic: here, a weakly supported clade of Chrysochromulinaceae and *Chrysocampanula* branched after Pavlomulinales and was sister to a moderately supported clade of the remaining haptophytes, with Prymnesiaceae I and Braarudosphaeraceae (moderate support) as sister groups at the base. Similarly, Prymnesiales were not monophyletic in the sequence-only ML subset tree: Chrysochromulinaceae formed a separate basal lineage to the Prymnesiophyceae, while a weakly supported clade of Prymnesiaceae was sister to the remaining coccolithophycean taxa (BS = (-/-/46)). Only in the sequence-structure ML subset tree were Prymnesiales depicted as monophyletic (BS = (47/-/55)) and in the same position as described in [7,14,44].

### Isochrysidales

The sister relationship between Isochrysidaceae and Noelaerhabdaceae, forming the Isochrysidales, has consistently received high or even full support in both previous studies and in the phylogenetic trees generated in this study. Depending on the taxon sampling, the immediate sister group of Isochrysidales reported in the literature has varied, including Coccolithales [14,42], a Coccolithales/Rhabdosphaeraceae clade [13], or clades comprising Coccolithales in other combinations [15,48,50]. Bootstrap support for these relationships also strongly depended on the dataset used. In studies with taxon sampling similar to ours, where both Zygodiscales and Syracosphaerales were included, the nodes defining the phylogenetic position of Isochrysidales often exhibited low or no bootstrap support [13,50]. In our analyses, the phylogenetic position of Isochrysidales varied slightly across different trees. Although there was a tendency for Isochrysidales to cluster with *Tergestiella* in the PNJ trees, the bootstrap values at these key nodes were generally low in the sequence-only analyses and moderate in the sequence-structure analyses, making it difficult to draw definitive conclusions about their exact phylogenetic placement. In contrast, the inclusion of Isochrysidales within the Calcihaptophyceae was strongly supported in both our study and previous literature.

### Coccolithales

Coccolithales was consistently recovered as a monophyletic and highly supported clade across all phylogenetic trees generated in this study. Previous research has identified close relationships between Coccolithales, Isochrysidales, and Syracosphaerales (primarily Rhabdosphaeraceae) [13–15,48,50]. However, the corresponding bootstrap values for these relationships have generally been insufficient to conclusively resolve their positions [14,15,48,50]. In our analyses, Coccolithales frequently appeared as sister to a Syracosphaerales/Zygodiscales clade, a relationship that was highly supported in the sequence-structure PNJ tree but less so in the sequence-only subtree (BS = 30/-/64). The sequence-only PNJ tree depicted a moderately supported Isochrysidales/Syracosphaerales/*Tergestiella*/Zygodiscales clade as sister to Coccolithales, while in the sequence-structure subtree, the same sister clade also included Braarudosphaeraceae, though with minimal support. Nevertheless, the overall relatedness between this clade and coccolithophores was strongly supported.

A consistently well-supported relationship observed in all trees, as well as in previous studies [14,15,48], was the Hymenomonadaceae/Chrysotilaceae clade. Calyptrosphaeraceae typically formed a distinct lineage, often as sister to the moderately to highly supported Calcidiscaceae/Coccolithaceae clade. This position is corroborated by SSU rDNA analyses [47]. In contrast, our sequence-only overview and both PNJ trees placed *Cruciplacolithus* (Coccolithaceae II) as sister to Calyptrosphaeraceae, albeit with low support—a relationship also reported in other 18S sequence-only studies [15,48], where bootstrap support for the Calyptrosphaeraceae/*Cruciplacolithus* clade was similarly low [48]. Consistent with previous findings [15,47,48], Coccolithaceae was never recovered as monophyletic, instead forming a clade with calcidiscacean taxa.

A particularly noteworthy aspect of recent research, especially in studies with taxon sampling similar to ours, is the phylogenetic position of *Hayaster perplexus* [48]. Although *Hayaster perplexus* is morphologically similar to Calcidiscaceae species [48,51] and is traditionally classified within this family, molecular data have suggested a closer relationship to Coccolithaceae. This has led to proposals for merging Coccolithaceae and Calcidiscaceae into a single family [48]. In our sequence-only trees, we observed a similar pattern of intermingling between coccolithacean and calcidiscacean species, with moderate to high support. However, the sequence-structure analyses consistently recovered *Hayaster perplexus* as sister to the calcidiscacean haptophytes, resulting in a monophyletic Calcidiscaceae clade. Specifically, in the sequence-structure PNJ tree, Calcidiscaceae (including *Hayaster perplexus*) formed a monophyletic group (BS = 83) that was sister to Coccolithaceae I (BS = 96), a relationship more strongly supported than the Calcidiscaceae I/Coccolithaceae I clade (BS = 74) with *Hayaster perplexus* at its base (BS = 77) as depicted in the sequence-only PNJ tree. The sequence-structure ML method provided only moderate support for Calcidiscaceae monophyly (BS = 53/-/39), whereas the sequence-only Maximum Likelihood analysis yielded higher support for the alternative tree structure. Notably, *Hayaster perplexus* was found even deeper within Calcidiscaceae in the sequence-structure subset analysis, as sister to *Calcidiscus* and *Oolithus*, with *Umbilicosphaera* at the base of the clade.

Taken together, our results indicate that, in contrast to sequence-only approaches (as used in [48]), the sequence-structure method supports the current classification of *Hayaster perplexus* as a calcidiscacean organism, in agreement with morphological evidence. Nevertheless, further research using additional molecular markers is warranted, as the bootstrap values from the ML analyses were not sufficient to draw definitive conclusions. Overall, our findings suggest that the relationships among families within Coccolithales are influenced by the phylogenetic method employed, underscoring the need for continued investigation in this area.

### Syracosphaerales and Zygodiscales

Consistent with previous studies [13,15,48,50], none of our analyses recovered Syracosphaerales as a monophyletic group. However, in all trees except the sequence-structure overview tree, the families assigned to Syracosphaerales consistently formed a clade together with the monophyletic Zygodiscales, which includes Helicosphaeraceae and Pontosphaeraceae. While this combined clade received moderate to high bootstrap support in the sequence-only trees, the sequence-structure trees provided weaker support for these relationships.

In the sequence-structure overview tree, a clade comprising Syracosphaeraceae and Zygodiscales was found to be sister to a Rhabdosphaeraceae/Coccolithales clade—a topology that is also frequently reported in the literature [15,48,50]. In contrast, the sequence-only overview and subtree analyses, as well as the sequence-structure PNJ tree, placed Coccolithales as the sister group to the entire Syracosphaerales/Zygodiscales clade. Notably, in the sequence-only PNJ tree, the clade was instead sister to an Isochrysidales/*Tergestiella* clade, which, in the sequence-structure subtree, also included Braarudosphaeraceae.

These alternative topologies more closely resemble the relationships reported by Edvardsen et al. [13], who found Zygodiscales forming a clade with Braarudosphaeraceae, which was sister to a clade where Syracosphaerales (likely represented by Syracosphaeraceae) occupied a basal position. Within this arrangement, Isochrysidales was sister to a Rhabdosphaeraceae/Coccolithales clade.

Unfortunately, neither our analyses nor those in previous studies provided strong bootstrap support for any particular phylogenetic placement of Syracosphaerales and Zygodiscales. The consistent lack of monophyly for Syracosphaerales across all trees suggests that the assumed close relationship between Rhabdosphaeraceae and Syracosphaeraceae should be reconsidered.

### Braarudosphaeraceae

In both overview and sequence-structure PNJ trees, Braarudosphaeraceae were recovered as sister to Prymnesiaceae I (Prymnesiales I), with moderate support in the PNJ tree, and thus placed within the non-calcifying haptophyte group Prymnesiales. A similar arrangement was reported in Hagino et al. [52]. In the sequence-only PNJ tree, Braarudosphaeraceae were sister to all Prymnesiales, though with low support. This uncertainty regarding the relationship between Braarudosphaeraceae and Prymnesiales, as seen in the PNJ trees, was also reflected in the Maximum Likelihood trees. There, Braarudosphaeraceae were sister to *Tergestiella* with high support, slightly higher in the sequence-structure approach compared to the sequence-only method. In the sequence-only subtree, the Braarudosphaeraceae/*Tergestiella* clade was sister to a Coccolithales/Syracosphaerales/Zygodiscales clade (BS = (27/-/45)). In contrast, in the sequence-structure method, the direct sister clade was Isochrysidales (BS = (74/-/44)). In both ML tree configurations, Braarudosphaeraceae were placed within Calcihaptophycidae, consistent with most studies, which position Braarudosphaeraceae as closely related to Isochrysidales [52]. Depending on taxon sampling or marker choice, Braarudosphaeraceae were recovered as sister to genera such as *Chrysoculter* or *Tergestiella* within the clade [47,49].

### ITS2

ITS2 sequence data often present significant alignment challenges due to length variations, which can result in alignment artifacts. Additionally, the availability of ITS2 sequences for haptophytes is limited and does not adequately represent the diversity within this group. Although previous studies have utilized ITS1 as a marker at the genus level [53], no comprehensive phylogenetic analyses of haptophytes using ITS2 as a marker have been conducted to date. In our sequence-only analyses, we encountered similar issues, with alignment artifacts and poor sequence congruence leading to phylogenetic trees that did not reflect established haptophyte relationships. For example, in the NJ overview tree, bootstrap analysis was not feasible due to extensive polytomies that prevented backbone resolution.

To address these issues, we incorporated secondary structure information into the sequence data and performed sequence-structure alignments. This approach markedly improved alignment quality, reduced artifacts, and produced phylogenetic trees more consistent with established 18S-based phylogenies and published literature. The ITS2 secondary structures obtained for seven haptophyte genera were generally consistent with known eukaryotic ITS2 features. For instance, *Isochrysis* and *Coccolithus* exhibited the canonical four-helix structure, while other genera displayed additional features such as a fifth helix or a sidearm on the third helix, as also observed in some other eukaryotes.

In the sequence-structure PNJ tree, the four-helix structures of *Coccolithus* and *Isochrysis* tended to cluster together. Notably, *Chrysotila*, characterized by an unusually long ITS2 sequence with a fifth helix and a sidearm, also grouped within this clade, which is consistent with current phylogenetic understanding.

Overall, each ITS2 secondary structure exhibited distinct characteristics, highlighting considerable diversity within the haptophyte clade. This structural variability, however, introduces a new challenge for using ITS2 as a phylogenetic marker in haptophytes. In our study, despite employing homology modeling with seven templates, many haptophyte genera had to be excluded from the final dataset due to insufficient homology. Consequently, if a broader range of ITS2 sequences were available, even more structures would need to be reconstructed for each genus, likely resulting in unresolved tree backbones.

In summary, unlike 18S rDNA, ITS2 may not be a robust marker for resolving deep phylogenetic relationships among haptophytes. However, due to its high variability, ITS2 remains a valuable tool for investigating relationships among closely related haptophyte genera and is particularly well suited for DNA barcoding applications.

## Conclusion

In this study, bootstrap values obtained from both 18S rDNA sequence-structure and sequence-only methods were consistently similar. Consequently, the increased robustness of phylogenetic analyses using sequence-structure information, as proposed by Keller et al. [1], could not be confirmed in our results. Nevertheless, in cases where sequence-only analyses and previous literature reported low support for certain nodes, the sequence-structure approach often suggested alternative phylogenetic placements. While these alternatives were not always better supported statistically, they provided new perspectives on the possible relationships among some groups. A notable example is the classification of *Hayaster perplexus*, where the sequence-structure method aligned with morphological observations, in contrast to the sequence-only approach.

Furthermore, in some nodes, the sequence-structure method produced phylogenies more consistent with the literature than those derived from sequence-only data. This is particularly remarkable given that most previous studies relied exclusively on sequence-only analyses. Our findings suggest that differences in taxon sampling likely have the greatest influence on phylogenetic outcomes between studies. This variability complicates direct comparisons, as many other studies included unclassified organisms or excluded certain species and entire haptophyte groups that were incorporated here. Additional factors affecting comparability include the use of partial 18S rDNA sequences or the combination of different genetic markers (such as 18S and 28S rDNA) in other studies. In contrast, our analyses were based exclusively on nearly complete 18S rDNA sequences, which are necessary for reconstructing secondary structure information.

Despite these methodological differences, our 18S rDNA sequence-structure phylogenies often agreed with widely accepted views of haptophyte relationships at broad taxonomic levels. In contrast, ITS2 sequences did not perform well for resolving higher-level phylogeny but proved useful for analyses at finer taxonomic scales. The secondary structure has clearly helped to minimize artifacts in the alignment.

Given that the sequence-structure approach is considered more robust and accurate than sequence-only analysis—supported by the simulations of Keller et al. [1]—and that Maximum Likelihood methods generally outperform Neighbor-Joining, we propose that the most reliable depiction of haptophyte phylogeny from this study is represented by the 18S sequence-structure Maximum Likelihood subset tree (Fig 3). The inferred backbone topology is as follows: (((((((((Braarudosphaeraceae, Tergestiella), Isochrysidales), ((Rhabdosphaeraceae, Zygodiscales), Syracosphaerales I)), Coccolithales), Prymnesiales), Phaeocystales), Pavlomulinales), Pavlovales), Centrohelida). Although this arrangement is broadly consistent with previous literature, the bootstrap support remains insufficient to fully resolve the backbone of haptophyte phylogeny with confidence.

## Supporting information

S1 Table List of all 18S and ITS2 rDNA sequences used or discarded in this study. (**A)** 18S rDNA sequences used or discarded – separated by a line. GenBank accession numbers and the respective organisms name (obtained from NCBI taxonomy or from the literature) as well as the percentage of structural transfer each organism received during homology modeling are displayed. For the discarded sequences also a column showing the reason for discarding is shown. Quotation marks (“) stand for “too short”. **(B)** ITS2 rDNA sequences used or discarded – separated by a line. GenBank accession numbers, the respective organisms name (obtained from NCBI taxonomy or from the literature), the structural transfer from homology modeling as well as the template used for each organism are shown. For the discarded sequences also a column showing the reason for discarding is shown. Quotation marks (“) signify the reason “too short”, a slash (/) the reason of too low structural transfer.
(DOCX)

S2 Table Summary of the most important results for the NJ-overview, the PNJ-original and the ML-subset trees compared to each other. **(A)** Table showing the most interesting results of comparing all 18S sequence-only and sequence-structure trees to each other. **(B)** Table showing the most interesting results of comparing all ITS2 sequence-only and sequence-structure trees to each other.
(DOCX)

S1 Fig Neighbor-Joining (NJ) tree based on 18S rDNA sequence-only data for 396 haptophytes and two centrohelids (outgroup). The sequence-only alignment was generated using ClustalX. Species names are accompanied by their respective GenBank accession numbers. According to (Edvardsen et al. 2011, Piwosz et al. 2019, Larsen 1999, Archontikis et al. 2023, Andersen et al. 2014, Bendif et al. 2013, Andersen et al. 2014) some species are renamed in contrast to NCBI taxonomy (cf. S1 Table). Clades selected for subsequent profile Neighbor-Joining (PNJ) analyses are alternately colored, and sequences used in later subset analyses are highlighted in bold. Families and orders within the haptophytes are labeled **A**–**T** as follows: **A**: Pavlovaceae, **B**: Prymnesiaceae I, **C**: Braarudosphaeraceae, **D**: Chrysochromulinaceae, **E**: Prymnesiaceae II, **F**: Chrysotilaceae, **G**: Hymenomonadaceae, **H**: Calcidiscaceae I, **I**: *Hayaster perplexus* (Calcidiscaceae II), **J**: Coccolithaceae I, **K**: Coccolithaceae II, **L**: Calyptrosphaeraceae, **M**: Rhabdosphaeraceae, **N**: Zygodiscales (including Pontosphaeraceae & Helicosphaeraceae), **O**: Syracosphaeraceae, **P**: *Tergestiella adriatica* (Watznaueriaceae), **Q**: Noelaerhabdaceae, **R**: Isochrysidaceae, **S**: Phaeocystaceae, **T**: Pavlomulinaceae. Orders are additionally annotated around the tree. The scale bar and branch lengths represent evolutionary distances.
(PDF)

S2 Fig Profile Neighbor-Joining (PNJ) tree based on 18S rDNA sequence-only profiles for 396 organisms. The tree was generated using ProfDistS, and the sequence-only alignment was generated using ClustalX. Species names are accompanied by their respective GenBank accession numbers. Two strains (GenBank accession AM490995, AB058358) were wrongly classified in NCBI (Edvardsen et al. 2011, Piwosz et al. 2019) and therefore renamed to Haptophyceae sp. (for outdated names see S1 Table). **A:** The cladogram on the left shows a three-times iterated PNJ tree. Bootstrap values from 100 pseudo-replicates are displayed at internal nodes. During each iteration, super-profiles were generated using existing profiles and bootstrap values >75. Numbers within the triangles indicate the number of sequences in manually defined profiles. Profile bootstrap values were derived and transferred from bootstrap testing (100 pseudo-replicates) performed on the overview NJ tree (S1 Fig). An asterisk (*) stands for a bootstrap value of 100. **B:** The phylogram on the right depicts the original PNJ tree without further iterations. Both trees are rooted with *Chlamydaster sterni* (GenBank accession AF534709) and *Sphaerastrum fockii* (GenBank accession AY749614). Branch lengths and the scale bar represent evolutionary distances. Colours indicate the corresponding haptophyte orders as follows: **blue**: Coccolithales, **green**: Syracosphaerales, **yellow**: Zygodiscales, **pink**: Isochrysidales, **violet**: Phaeocystales, **red**: Prymnesiales, **orange**: Pavlomulinales, **brown**: Pavlovales.
(PDF)

S3 Fig Maximum-likelihood (ML) tree based on 18S rDNA sequence-only data for a manually chosen subset of 53 organisms representing the profiles used in the PNJ analysis (S2 Fig). The tree was constructed in MEGA using the TN93 + G + I model. The sequence-only alignment was generated using ClustalX. Some species (cf. S1 Table) were named differently than in NCBI according to (Larsen 1999, Archontikis et al. 2023, Bendif et al. 2013, Andersen et al. 2014). Alternating colors highlight the PNJ profiles, and family and order names are displayed on the right side of the tree. Non-monophyletic orders are indicated by Roman numerals. The branch length of the outgroup (*Sphaerastrum fockii* and *Chlamydaster sterni*) is trimmed to 25%. Branch lengths and the scale bar represent evolutionary distances. Bootstrap values are shown for three methods—neighbor-joining (NJ, obtained in ProfDistS), maximum-parsimony (MP, obtained in PAUP), and maximum-likelihood (ML)—in the order NJ/MP/ML. A bootstrap value of 100 is marked with an asterisk (*), while a dash (-) indicates differing tree topologies across methods.
(PDF)

S4 Fig Boxplots showing the length distribution of ITS2 sequences for genera used in this study. The sequence lengths were obtained from all sequences used in the ITS2 analysis. The length distribution is shown for *Chrysochromulina* (turquois), *Chrysotila* (orange), *Coccolithus* (blue), *Dicrateria* (violet), *Isochrysis* (lightgreen), *Phaeocystis* (yello*w)* and *Prymnesium* (beige).
(PDF)

S5 Fig Neighbor-Joining (NJ) tree based on ITS2 rDNA sequence-only data for 137 organisms. The sequence-only alignment was generated using ClustalX. Species names are accompanied by their respective GenBank accession numbers. According to (Larsen 1999, Andersen et al. 2014, Bendif et al. 2013) species are renamed in contrast to NCBI taxonomy (cf. S1 Table). As the outgroup, Phaeocystales was chosen. Clades selected for subsequent profile Neighbor-Joining (PNJ) analyses are alternately colored, and sequences used in later subset analyses are highlighted in bold. Families and orders within Prymnesiophyceae are labeled **A**–**H** as follows: **A**: *Phaeocystis*, **B**: *Prymnesium*, **C**: *Dicrateria*, **D**: *Coccolithus*, **E**: *Chrysotila*, **F**: *Chrysochromulina I*, **G**: *Isochrysis,* ***H***: *Chrysochromulina andersonii.* Orders are additionally annotated around the tree. The scale bar and branch lengths represent evolutionary distances.
(PDF)

S6 Fig Profile Neighbour-Joining (PNJ) tree based on ITS2 rDNA sequence-only profiles for 137 organisms. The tree was generated using ProfDistS, and the sequence-only alignment was generated using ClustalX. **A**: The cladogram on the left shows a three-times iterated PNJ tree. Bootstrap values from 100 pseudo-replicates are displayed at internal nodes. During each iteration, super-profiles were generated using existing profiles and bootstrap values >75. Numbers within the triangles indicate the number of sequences in manually defined profiles. Profile bootstrap values could not be obtained due to polytomy. **B**: The phylogram on the right depicts the original PNJ tree without further iterations. Both trees are rooted with Phaeocystales. Branch lengths and the scale bar represent evolutionary distances. Colours indicate the corresponding haptophyte orders as follows: **blue**: Coccolithales, **pink**: Isochrysidales, **violet**: Phaeocystales, **red**: Prymnesiales.
(PDF)

S7 Fig Maximum-likelihood (ML) tree based on ITS2 rDNA sequence-only data for a manually chosen subset of 17 organisms representing the profiles used in the PNJ analysis. The tree was constructed in MEGA using the HKY + G model. The sequence-only alignment was generated using ClustalX. According to (Andersen et al. 2014, Larsen 1999) species are renamed in contrast to NCBI taxonomy (cf. S1 Table). Alternating colors highlight the PNJ profiles. Genera and order names are displayed on the right side of the tree. Non-monophyletic orders are indicated by Roman numerals. Phaeocystales was chosen as outgroup. Branch lengths and the scale bar represent evolutionary distances. Bootstrap values are shown for three methods—neighbor-joining (NJ, obtained in MEGA using the Maximum-Composite-Likelihood+G model as replacement for HKY + G), maximum-parsimony (MP, obtained in PAUP), and maximum-likelihood (ML)—in the order NJ/MP/ML. A bootstrap value of 100 is marked with an asterisk (*), while a dash (-) indicates differing tree topologies across methods.
(PDF)

S1 File 18S sequence-structure alignment (overview). (TXT)

S2 File 18S sequence-only alignment (overview). (TXT)

S3 File 18S sequence-structure alignment (subset). (TXT)

S4 File 18S sequence-only alignment (subset). (TXT)

S5 File ITS2 sequence-structure alignment (overview). (TXT)

S6 File ITS2 sequence-only alignment (overview). (TXT)

S7 File ITS2 sequence-structure alignment (subset). (TXT)

S8 File ITS2 sequence-only alignment (subset). (TXT)
