# Supplementary material for: ITS2 and 18S rRNA gene sequence-structure phylogeny of the Haptophyta (Haptista)
Source: PLoS One. 2026 Mar 19;21(3):e0344353. doi: 10.1371/journal.pone.0344353 (PMC13001949; doi:10.1371/journal.pone.0344353)
Supplement: S1 Table — (A) 18S rDNA sequences used or discarded – separated by a line. GenBank accession numbers and the respective organisms name (obtained from NCBI taxonomy or from the literature) as well as the percentage of structural transfer each organism received during homology modeling are displayed. For the discarded sequences also a column showing the reason for discarding is shown. Quotation marks (“) stand for “too short”. (B) ITS2 rDNA sequences used or discarded – separated by a line. GenBank accession numbers, the respective organisms name (obtained from NCBI taxonomy or from the literature), the structural transfer from homology modeling as well as the template used for each organism are shown. For the discarded sequences also a column showing the reason for discarding is shown. Quotation marks (“) signify the reason “too short”, a slash (/) the reason of too low structural transfer. (DOCX) [file pone.0344353.s001.docx]

**(A)**: 18S rDNA sequences used (above) and discarded (below) separated by a thick line.

| **Genbank**  **accession** | **Species name**  **(obtained from literature)** | **Outdated species name**  **(obtained from NCBI)** | **Helix Transfer**  **(in %)** |
| --- | --- | --- | --- |
| AM490985 | *Algirosphaera robusta* |  | 97.082 |
| AB478414 | *Braarudosphaera bigelowii* |  | 97.878 |
| AB478412 | *Braarudosphaera bigelowii* |  | 97.878 |
| AB250785 | *Braarudosphaera bigelowii* |  | 98.143 |
| AB250784 | *Braarudosphaera bigelowii* |  | 98.143 |
| AB778293 | *Braarudosphaera bigelowii* |  | 98.143 |
| AB847975 | *Braarudosphaera bigelowii* |  | 98.143 |
| AB847973 | *Braarudosphaera bigelowii* |  | 97.613 |
| AB847972 | *Braarudosphaera bigelowii* |  | 98.143 |
| AB847971 | *Braarudosphaera bigelowii* |  | 97.082 |
| AB847970 | *Braarudosphaera bigelowii* |  | 98.143 |
| AB847981 | *Braarudosphaera bigelowii* |  | 98.143 |
| AB847980 | *Braarudosphaera bigelowii* |  | 98.143 |
| AB847978 | *Braarudosphaera bigelowii* |  | 98.143 |
| AB847977 | *Braarudosphaera bigelowii* |  | 98.143 |
| AB847976 | *Braarudosphaera bigelowii* |  | 98.143 |
| AB847974 | *Braarudosphaera bigelowii* |  | 97.878 |
| AM490994 | *Braarudosphaera bigelowii* | *Chrysochromulina parkae* | 97.979 |
| KF422619 | *Calcidiscus leptoporus* |  | 97.613 |
| AJ544116 | *Calcidiscus leptoporus* |  | 97.613 |
| AJ544115 | *Calcidiscus quadriperforatus* |  | 97.613 |
| AM491024 | *Calyptrosphaera radiata* |  | 97.613 |
| AM490990 | *Calyptrosphaera sphaeroidea* |  | 97.613 |
| AF534709 | *Chlamydaster sterni* |  | 88.859 |
| AM491022 | *Chrysocampanula spinifera* |  | 97.613 |
| KY980155 | *Chrysocampanula spinifera* |  | 98.408 |
| KY980148 | *Chrysocampanula spinifera* |  | 98.408 |
| KY980136 | *Chrysocampanula spinifera* |  | 98.408 |
| KY980116 | *Chrysocampanula spinifera* |  | 98.408 |
| KY980110 | *Chrysocampanula spinifera* |  | 97.347 |
| KY980108 | *Chrysocampanula spinifera* |  | 97.878 |
| KY980104 | *Chrysocampanula spinifera* |  | 98.143 |
| KY980095 | *Chrysocampanula spinifera* |  | 97.347 |
| KY980092 | *Chrysocampanula spinifera* |  | 98.408 |
| KY980081 | *Chrysocampanula spinifera* |  | 98.143 |
| KY980075 | *Chrysocampanula spinifera* |  | 98.143 |
| KY980074 | *Chrysocampanula spinifera* |  | 98.408 |
| KY980151 | *Chrysocampanula spinifera* |  | 98.408 |
| AJ246278 | *Chrysochromulina acantha* |  | 96.021 |
| FN599059 | *Chrysochromulina acantha* |  | 96.286 |
| AJ246273 | *Chrysochromulina campanulifera* |  | 96.552 |
| AM491018 | *Chrysochromulina cymbium* |  | 96.021 |
| AM491017 | *Chrysochromulina leadbeateri* |  | 96.286 |
| ON815373 | *Chrysochromulina leadbeateri* |  | 97.613 |
| ON815372 | *Chrysochromulina leadbeateri* |  | 97.613 |
| AM491019 | *Chrysochromulina parva* |  | 94.695 |
| AM491025 | *Chrysochromulina rotalis* |  | 95.491 |
| AJ246274 | *Chrysochromulina scutellum* |  | 97.347 |
| AM491021 | *Chrysochromulina simplex* |  | 97.082 |
| KY980316 | *Chrysochromulina strobilus* |  | 97.082 |
| KY980056 | *Chrysochromulina strobilus* |  | 96.817 |
| KY980032 | *Chrysochromulina strobilus* |  | 96.817 |
| KY979987 | *Chrysochromulina strobilus* |  | 96.552 |
| FN599060 | *Chrysochromulina strobilus* |  | 96.552 |
| KY980030 | *Chrysochromulina strobilus* |  | 96.817 |
| AJ246277 | *Chrysochromulina throndsenii* |  | 95.491 |
| AJ246279 | *Chrysochromulina throndsenii* |  | 95.491 |
| AJ246263 | *Chrysotila carterae* |  | 97.347 |
| AJ544120 | *Chrysotila carterae* |  | 97.082 |
| MG022757 | *Chrysotila carterae* |  | 97.347 |
| HQ877918 | *Chrysotila carterae* |  | 97.082 |
| AJ544121 | *Chrysotila dentata* |  | 97.347 |
| KJ756811 | *Chrysotila dentata* |  | 97.347 |
| AM936924 | *Chrysotila elongata* | *Pleurochrysis elongata* | 96.552 |
| AJ246264 | *Chrysotila elongata* | *Pleurochrysis elongata* | 95.225 |
| AM490972 | *Chrysotila gayraliae* |  | 97.347 |
| AM490977 | *Chrysotila placolithoides* |  | 97.347 |
| AM490973 | *Chrysotila pseudoroscoffensis* |  | 97.347 |
| MW471017 | *Chrysotila pseudoroscoffensis* |  | 97.247 |
| MG022748 | *Chrysotila pseudoroscoffensis* |  | 97.347 |
| AM490974 | *Chrysotila roscoffensis* |  | 97.347 |
| AM490978 | *Chrysotila scherffelii* |  | 97.347 |
| KF728655 | *Chrysotila stipitata* |  | 97.347 |
| KF728654 | *Chrysotila stipitata* |  | 97.347 |
| KF728653 | *Chrysotila stipitata* |  | 97.347 |
| KF696663 | *Chrysotila stipitata* |  | 97.347 |
| AJ544117 | *Coccolithus braarudii* |  | 97.878 |
| AJ246261 | *Coccolithus pelagicus* |  | 97.878 |
| AM490986 | *Coronosphaera mediterranea* |  | 97.613 |
| AJ246262 | *Cruciplacolithus neohelis* |  | 98.143 |
| AB058348 | *Cruciplacolithus neohelis* |  | 97.878 |
| JF714242 | *Diacronema ennorea* |  | 95.491 |
| KY980093 | *Diacronema ennorea* |  | 96.817 |
| AJ515247 | *Diacronema lutheri* |  | 94.164 |
| AF106053 | *Diacronema lutheri* |  | 93.369 |
| JF489960 | *Diacronema lutheri* |  | 93.369 |
| HQ877906 | *Diacronema lutheri* |  | 94.164 |
| HQ877905 | *Diacronema lutheri* |  | 94.164 |
| HQ877904 | *Diacronema lutheri* |  | 93.899 |
| JF714238 | *Diacronema lutheri* |  | 94.164 |
| JF714236 | *Diacronema lutheri* |  | 94.164 |
| MN791087 | *Diacronema lutheri* |  | 94.430 |
| PQ870413 | *Diacronema lutheri* |  | 94.960 |
| MG022753 | *Diacronema lutheri* |  | 95.225 |
| AF102369 | *Diacronema lutheri* |  | 93.634 |
| JF489961 | *Diacronema lutheri* |  | 93.634 |
| HQ877907 | *Diacronema lutheri* |  | 94.164 |
| DQ207406 | *Diacronema noctivaga* | *Pavlova noctivaga* | 96.021 |
| MK834581 | *Diacronema noctivaga* | *Pavlova noctivaga* | 95.225 |
| JF714243 | *Diacronema noctivaga* | *Pavlova noctivaga* | 96.021 |
| JF714222 | *Diacronema noctivaga* | *Pavlova noctivaga* | 96.021 |
| AJ515248 | *Diacronema virescens* | *Pavlova virescens* | 94.164 |
| JF714235 | *Diacronema virescens* | *Pavlova virescens* | 94.164 |
| DQ075201 | *Diacronema viridis* |  | 94.430 |
| HQ877913 | *Diacronema viridis* |  | 94.430 |
| AJ515246 | *Diacronema vlkianum* |  | 94.695 |
| AF106056 | *Diacronema vlkianum* |  | 93.103 |
| HF678450 | *Diacronema vlkianum* |  | 93.369 |
| JF714241 | *Diacronema vlkianum* |  | 94.695 |
| AJ246267 | *Dicrateria rotunda* |  | 96.817 |
| LC519889 | *Dicrateria rotunda* |  | 97.613 |
| KT861304 | *Dicrateria rotunda* |  | 97.613 |
| AM491014 | *Dicrateria rotunda* |  | 97.613 |
| DQ531625 | *Exanthemachrysis gayraliae* |  | 93.634 |
| AJ515250 | *Exanthemachrysis gayraliae* |  | 94.164 |
| AF106060 | *Exanthemachrysis gayraliae* |  | 94.695 |
| AF106050 | *Exanthemachrysis gayraliae* |  | 93.634 |
| AF106048 | *Exanthemachrysis gayraliae* |  | 94.695 |
| JF714223 | *Exanthemachrysis gayraliae* |  | 93.899 |
| AF102372 | *Exanthemachrysis gayraliae* |  | 94.695 |
| KX056532 | *Gephyrocapsa ericsonii* |  | 97.082 |
| KX229688 | *Gephyrocapsa huxleyi* | *Emiliania huxleyi* | 97.347 |
| KC404141 | *Gephyrocapsa huxleyi* | *Emiliania huxleyi* | 97.347 |
| KC404140 | *Gephyrocapsa huxleyi* | *Emiliania huxleyi* | 97.347 |
| KC404139 | *Gephyrocapsa huxleyi* | *Emiliania huxleyi* | 97.347 |
| KC404138 | *Gephyrocapsa huxleyi* | *Emiliania huxleyi* | 97.347 |
| KC404137 | *Gephyrocapsa huxleyi* | *Emiliania huxleyi* | 97.347 |
| KT861255 | *Gephyrocapsa huxleyi* | *Emiliania huxleyi* | 97.347 |
| KC404136 | *Gephyrocapsa huxleyi* | *Emiliania huxleyi* | 97.347 |
| KC404134 | *Gephyrocapsa huxleyi* | *Emiliania huxleyi* | 97.347 |
| KC404131 | *Gephyrocapsa huxleyi* | *Emiliania huxleyi* | 97.347 |
| KC404129 | *Gephyrocapsa huxleyi* | *Emiliania huxleyi* | 97.347 |
| KC404128 | *Gephyrocapsa huxleyi* | *Emiliania huxleyi* | 97.347 |
| KC404127 | *Gephyrocapsa huxleyi* | *Emiliania huxleyi* | 97.347 |
| KC404126 | *Gephyrocapsa huxleyi* | *Emiliania huxleyi* | 97.347 |
| KC404125 | *Gephyrocapsa huxleyi* | *Emiliania huxleyi* | 97.347 |
| KC404124 | *Gephyrocapsa huxleyi* | *Emiliania huxleyi* | 97.347 |
| KC404123 | *Gephyrocapsa huxleyi* | *Emiliania huxleyi* | 97.347 |
| KC404122 | *Gephyrocapsa huxleyi* | *Emiliania huxleyi* | 97.347 |
| KC404121 | *Gephyrocapsa huxleyi* | *Emiliania huxleyi* | 97.347 |
| KC404120 | *Gephyrocapsa huxleyi* | *Emiliania huxleyi* | 97.347 |
| HQ877901 | *Gephyrocapsa huxleyi* | *Emiliania huxleyi* | 97.347 |
| MN824007 | *Gephyrocapsa huxleyi* | *Emiliania huxleyi* | 97.082 |
| AF184167 | *Gephyrocapsa huxleyi* | *Emiliania huxleyi* | 97.347 |
| L04957 | *Gephyrocapsa huxleyi* | *Emiliania huxleyi* | 97.347 |
| M87327 | *Gephyrocapsa huxleyi* | *Emiliania huxleyi* | 97.347 |
| KC404135 | *Gephyrocapsa huxleyi* | *Emiliania huxleyi* | 97.347 |
| KC404133 | *Gephyrocapsa huxleyi* | *Emiliania huxleyi* | 97.347 |
| KC404132 | *Gephyrocapsa huxleyi* | *Emiliania huxleyi* | 97.347 |
| KC404130 | *Gephyrocapsa huxleyi* | *Emiliania huxleyi* | 97.347 |
| KP282839 | *Gephyrocapsa muellerae* |  | 97.347 |
| KP282838 | *Gephyrocapsa muellerae* |  | 97.347 |
| KP282837 | *Gephyrocapsa muellerae* |  | 97.347 |
| AJ246276 | *Gephyrocapsa oceanica* |  | 97.347 |
| AB183665 | *Gephyrocapsa oceanica* |  | 97.347 |
| KC404159 | *Gephyrocapsa oceanica* |  | 97.347 |
| KC404158 | *Gephyrocapsa oceanica* |  | 97.347 |
| KC404157 | *Gephyrocapsa oceanica* |  | 97.347 |
| KC404156 | *Gephyrocapsa oceanica* |  | 97.347 |
| KC404155 | *Gephyrocapsa oceanica* |  | 97.347 |
| KC404154 | *Gephyrocapsa oceanica* |  | 97.347 |
| KC404153 | *Gephyrocapsa oceanica* |  | 97.347 |
| KC404151 | *Gephyrocapsa oceanica* |  | 97.347 |
| KC404150 | *Gephyrocapsa oceanica* |  | 97.347 |
| KC404147 | *Gephyrocapsa oceanica* |  | 97.347 |
| KC404145 | *Gephyrocapsa oceanica* |  | 97.347 |
| KC404144 | *Gephyrocapsa oceanica* |  | 97.347 |
| KC404143 | *Gephyrocapsa oceanica* |  | 97.347 |
| KC404152 | *Gephyrocapsa oceanica* |  | 97.347 |
| KC404149 | *Gephyrocapsa oceanica* |  | 97.347 |
| KC404148 | *Gephyrocapsa oceanica* |  | 97.347 |
| AB058360 | *Gephyrocapsa oceanica* |  | 97.347 |
| KC404146 | *Gephyrocapsa oceanica* |  | 97.347 |
| KC404142 | *Gephyrocapsa oceanica* |  | 97.347 |
| KX056536 | *Gephyrocapsa parvula* |  | 97.347 |
| KX056535 | *Gephyrocapsa parvula* |  | 97.347 |
| KX056534 | *Gephyrocapsa parvula* |  | 97.347 |
| KX056533 | *Gephyrocapsa parvula* |  | 97.082 |
| AM491012 | *Haptolina brevifila* |  | 97.347 |
| AM491011 | *Haptolina* cf. *herdlensis* |  | 97.613 |
| AB058369 | *Haptolina ericina* |  | 97.878 |
| AM491030 | *Haptolina ericina* |  | 96.817 |
| AM491013 | *Haptolina fragaria* |  | 98.143 |
| AJ246272 | *Haptolina hirta* |  | 96.552 |
| AB058358 | *Haptophyceae* sp. | *Haptolina brevifila* | 97.878 |
| AM490995 | *Haptophyceae* sp. | *Haptolina brevifila* | 97.613 |
| LC771592 | *Hayaster perplexus* |  | 97.613 |
| KF422620 | *Helicosphaera carteri* |  | 98.143 |
| AM490983 | *Helicosphaera carteri* |  | 98.143 |
| AM490982 | *Hymenomonas coronata* |  | 98.143 |
| AM490981 | *Hymenomonas globosa* |  | 98.408 |
| HQ877911 | *Isochrysis* aff*. galbana* |  | 97.347 |
| HQ877902 | *Isochrysis* aff. *galbana* |  | 97.082 |
| OR178772 | *Isochrysis galbana* |  | 97.082 |
| HM149542 | *Isochrysis galbana* |  | 96.552 |
| HM149540 | *Isochrysis galbana* |  | 96.021 |
| GQ118682 | *Isochrysis galbana* |  | 97.347 |
| AJ246266 | *Isochrysis galbana* |  | 96.552 |
| MH182066 | *Isochrysis galbana* |  | 97.347 |
| KY054965 | *Isochrysis galbana* |  | 97.347 |
| KX980525 | *Isochrysis galbana* |  | 97.347 |
| KX980523 | *Isochrysis galbana* |  | 97.347 |
| KX980522 | *Isochrysis galbana* |  | 97.347 |
| KX980521 | *Isochrysis galbana* |  | 97.347 |
| KM057843 | *Isochrysis galbana* |  | 94.960 |
| MH166731 | *Isochrysis galbana* |  | 97.347 |
| KC594686 | *Isochrysis galbana* |  | 94.164 |
| JF489946 | *Isochrysis galbana* |  | 96.552 |
| JF489945 | *Isochrysis galbana* |  | 97.347 |
| HM246242 | *Isochrysis galbana* |  | 97.347 |
| HM149543 | *Isochrysis galbana* |  | 97.347 |
| PQ870412 | *Isochrysis galbana* |  | 97.082 |
| MG022756 | *Isochrysis galbana* |  | 97.082 |
| MG022752 | *Isochrysis galbana* |  | 97.082 |
| HM149541 | *Isochrysis galbana* |  | 97.082 |
| KX980524 | *Isochrysis galbana* |  | 97.082 |
| JF489947 | *Isochrysis galbana* |  | 97.347 |
| HQ877910 | *Isochrysis galbana* |  | 97.347 |
| HQ877903 | *Isochrysis galbana* |  | 97.347 |
| AM490996 | *Isochrysis litoralis* |  | 97.082 |
| HQ877921 | *Isocrysis galbana* | *Pseudoisochrysis paradoxa* | 96.817 |
| HF678451 | *Isocrysis galbana* | *Pseudoisochrysis paradoxa* | 97.082 |
| AM490999 | *Isocrysis galbana* | *Pseudoisochrysis paradoxa* | 97.082 |
| AM490979 | *Jomonlithus littoralis* |  | 98.143 |
| FR865767 | *Ochrosphaera neapolitana* |  | 97.878 |
| OQ799058 | *Ochrosphaera neapolitana* |  | 97.878 |
| AM490980 | *Ochrosphaera verrucosa* |  | 97.878 |
| AM491026 | *Oolithotus fragilis* |  | 97.613 |
| MH182064 | *P. parvum f. parvum* | *Prymnesium parvum* | 98.674 |
| KJ756812 | *P. parvum f. parvum* | *Prymnesium parvum* | 98.674 |
| OR231183 | *P. parvum f. parvum* | *Prymnesium parvum* | 98.674 |
| MW471019 | *P. parvum f. parvum* | *Prymnesium parvum* | 98.674 |
| MN727031 | *P. parvum f. parvum* | *Prymnesium parvum* | 98.674 |
| PP660314 | *P. parvum f. parvum* | *Prymnesium parvum* | 98.674 |
| PP660313 | *P. parvum f. parvum* | *Prymnesium parvum* | 98.674 |
| PP660312 | *P. parvum f. parvum* | *Prymnesium parvum* | 98.674 |
| PP660311 | *P. parvum f. parvum* | *Prymnesium parvum* | 98.674 |
| MG022755 | *P. parvum f. parvum* | *Prymnesium parvum* | 98.674 |
| MG022754 | *P. parvum f. parvum* | *Prymnesium parvum* | 98.674 |
| AJ246269 | *P. parvum f. parvum* | *Prymnesium parvum* | 98.674 |
| LC599498 | *Pavlomulina ranunculiformis* | *Haptophyceae* sp. NIES-3900 | 98.939 |
| LC603171 | *Pavlomulina ranunculiformis* | *Haptophyta* sp. RCC3430 | 98.939 |
| JF714231 | *Pavlova granifera* |  | 91.777 |
| KF925344 | *Pavlova gyrans* |  | 93.634 |
| AF106055 | *Pavlova gyrans* |  | 93.369 |
| AF106054 | *Pavlova gyrans* |  | 93.369 |
| AF102371 | *Pavlova gyrans* |  | 93.634 |
| U40922 | *Pavlova gyrans* |  | 92.573 |
| JF714249 | *Pavlova gyrans* |  | 93.634 |
| JF714246 | *Pavlova gyrans* |  | 93.634 |
| FR865772 | *Pavlova gyrans* |  | 93.634 |
| FR865771 | *Pavlova gyrans* |  | 93.369 |
| HQ877917 | *Pavlova gyrans* |  | 93.634 |
| HQ877912 | *Pavlova gyrans* |  | 93.634 |
| AF106051 | *Pavlova gyrans* |  | 93.634 |
| AB293551 | *Pavlova pinguis* |  | 91.777 |
| AB183600 | *Pavlova pinguis* |  | 91.512 |
| AF106058 | *Pavlova pinguis* |  | 91.512 |
| AF106057 | *Pavlova pinguis* |  | 91.777 |
| AF106052 | *Pavlova pinguis* |  | 91.777 |
| AF106047 | *Pavlova pinguis* |  | 92.573 |
| AF102373 | *Pavlova pinguis* |  | 93.103 |
| AF102370 | *Pavlova pinguis* |  | 91.777 |
| JF714247 | *Pavlova pinguis* |  | 93.634 |
| HQ877914 | *Pavlova pinguis* |  | 90.981 |
| PV257734 | *Pavlova pinguis* |  | 91.777 |
| OP536011 | *Pavlova pinguis* |  | 93.103 |
| JF714248 | *Pavlova pinguis* |  | 93.369 |
| AJ515249 | *Pavlova pseudogranifera* |  | 91.777 |
| AB058367 | *Phaeocystis antarctica* |  | 98.143 |
| X77478 | *Phaeocystis antarctica* |  | 97.613 |
| X77477 | *Phaeocystis antarctica* |  | 97.347 |
| ON888443 | *Phaeocystis antarctica* |  | 98.143 |
| KF925339 | *Phaeocystis antarctica* |  | 98.143 |
| X77481 | *Phaeocystis antarctica* |  | 98.143 |
| X77479 | *Phaeocystis antarctica* |  | 98.143 |
| X77480 | *Phaeocystis antarctica* |  | 97.613 |
| JN381495 | *Phaeocystis antarctica* |  | 98.143 |
| JN381494 | *Phaeocystis antarctica* |  | 98.143 |
| JN381493 | *Phaeocystis antarctica* |  | 98.143 |
| AF163147 | *Phaeocystis cordata* |  | 97.347 |
| JX660992 | *Phaeocystis cordata* |  | 96.552 |
| GQ118981 | *Phaeocystis globosa* |  | 97.082 |
| GQ118980 | *Phaeocystis globosa* |  | 97.878 |
| EU024765 | *Phaeocystis globosa* |  | 97.878 |
| MT760788 | *Phaeocystis globosa* |  | 97.878 |
| AY851301 | *Phaeocystis globosa* |  | 96.817 |
| AY851300 | *Phaeocystis globosa* |  | 97.347 |
| AJ278035 | *Phaeocystis globosa* |  | 97.082 |
| EF100712 | *Phaeocystis globosa* |  | 97.613 |
| JX660994 | *Phaeocystis globosa* |  | 97.613 |
| JX660988 | *Phaeocystis globosa* |  | 97.878 |
| JX660987 | *Phaeocystis globosa* |  | 96.552 |
| EU127475 | *Phaeocystis globosa* |  | 97.878 |
| MW575289 | *Phaeocystis globosa* |  | 98.143 |
| MW575291 | *Phaeocystis globosa* |  | 98.143 |
| MW575290 | *Phaeocystis globosa* |  | 98.143 |
| MZ365004 | *Phaeocystis globosa* |  | 98.143 |
| PQ640342 | *Phaeocystis globosa* |  | 97.613 |
| PQ640287 | *Phaeocystis globosa* |  | 97.613 |
| OP531884 | *Phaeocystis globosa* |  | 97.878 |
| OP531886 | *Phaeocystis globosa* |  | 98.143 |
| OP531885 | *Phaeocystis globosa* |  | 98.143 |
| MN927500 | *Phaeocystis globosa* |  | 98.143 |
| MN917485 | *Phaeocystis globosa* |  | 98.143 |
| MN927483 | *Phaeocystis globosa* |  | 98.143 |
| MN927499 | *Phaeocystis globosa* |  | 98.143 |
| MN927498 | *Phaeocystis globosa* |  | 98.143 |
| MN927497 | *Phaeocystis globosa* |  | 98.143 |
| MN927496 | *Phaeocystis globosa* |  | 98.143 |
| MN927495 | *Phaeocystis globosa* |  | 98.143 |
| MN927494 | *Phaeocystis globosa* |  | 98.143 |
| MN927493 | *Phaeocystis globosa* |  | 98.143 |
| MN927492 | *Phaeocystis globosa* |  | 98.143 |
| MN927491 | *Phaeocystis globosa* |  | 98.143 |
| MN927490 | *Phaeocystis globosa* |  | 98.143 |
| MN927489 | *Phaeocystis globosa* |  | 98.143 |
| MN927488 | *Phaeocystis globosa* |  | 98.143 |
| MN927487 | *Phaeocystis globosa* |  | 98.143 |
| MN927486 | *Phaeocystis globosa* |  | 98.143 |
| MN927484 | *Phaeocystis globosa* |  | 98.143 |
| MN927482 | *Phaeocystis globosa* |  | 98.143 |
| MN927481 | *Phaeocystis globosa* |  | 98.143 |
| MN927480 | *Phaeocystis globosa* |  | 98.143 |
| MN927479 | *Phaeocystis globosa* |  | 98.143 |
| MN826700 | *Phaeocystis globosa* |  | 98.143 |
| MN826699 | *Phaeocystis globosa* |  | 98.143 |
| MN826697 | *Phaeocystis globosa* |  | 98.143 |
| MN826696 | *Phaeocystis globosa* |  | 98.143 |
| MN826695 | *Phaeocystis globosa* |  | 98.143 |
| MN826694 | *Phaeocystis globosa* |  | 98.143 |
| MN826693 | *Phaeocystis globosa* |  | 98.143 |
| MN826692 | *Phaeocystis globosa* |  | 98.143 |
| MG914020 | *Phaeocystis globosa* |  | 98.143 |
| AF182110 | *Phaeocystis globosa* |  | 98.143 |
| AF182109 | *Phaeocystis globosa* |  | 97.878 |
| AF182115 | *Phaeocystis globosa* |  | 97.878 |
| AF182113 | *Phaeocystis globosa* |  | 97.347 |
| AF182112 | *Phaeocystis globosa* |  | 98.143 |
| AF182111 | *Phaeocystis globosa* |  | 97.878 |
| GQ118979 | *Phaeocystis globosa* |  | 97.878 |
| MT760789 | *Phaeocystis globosa* |  | 97.878 |
| X77476 | *Phaeocystis globosa* |  | 97.347 |
| JX660986 | *Phaeocystis globosa* |  | 96.552 |
| MN826698 | *Phaeocystis globosa* |  | 97.878 |
| AF163148 | *Phaeocystis jahnii* |  | 97.878 |
| KR091066 | *Phaeocystis pouchetii* |  | 97.878 |
| AJ278036 | *Phaeocystis pouchetii* |  | 97.613 |
| X77475 | *Phaeocystis pouchetii* |  | 97.878 |
| AF182114 | *Phaeocystis pouchetii* |  | 97.878 |
| AM491007 | *Prymnesium annuliferum* |  | 99.469 |
| U40923 | *Prymnesium calathiferum* |  | 98.939 |
| AM491008 | *Prymnesium calathiferum* |  | 98.939 |
| AM491029 | *Prymnesium chiton* |  | 98.408 |
| AM491005 | *Prymnesium faveolatum* |  | 99.469 |
| AJ246271 | *Prymnesium kappa* |  | 98.408 |
| AM491010 | *Prymnesium minus* |  | 98.143 |
| AJ246268 | *Prymnesium nemamethecum* |  | 98.674 |
| AM491004 | *Prymnesium nemamethecum* |  | 98.674 |
| L34670 | *Prymnesium parvum* f. *patelliferum* |  | 98.408 |
| L34671 | *Prymnesium parvum* f. *patelliferum* |  | 98.674 |
| MW471018 | *Prymnesium parvum* f. *patelliferum* |  | 98.674 |
| KY054990 | *Prymnesium pienaarii* |  | 99.204 |
| KU561110 | *Prymnesium pienaarii* |  | 99.204 |
| AM491027 | *Prymnesium pienaarii* |  | 99.204 |
| AM491003 | *Prymnesium pigrum* |  | 99.204 |
| FR865770 | *Prymnesium pigrum* |  | 98.939 |
| AJ004866 | *Prymnesium polylepis* |  | 99.735 |
| KU561118 | *Prymnesium simplex* |  | 99.204 |
| AM491028 | *Prymnesium simplex* |  | 98.939 |
| AM491009 | *Prymnesium* sp. | *Prymnesium* cf. *polylepis* | 99.469 |
| AJ004868 | *Prymnesium sp.* | *Prymnesium* aff. *polylepis* | 99.469 |
| AM491001 | *Prymnesium zebrinum* |  | 99.204 |
| AM491016 | *Pseudohaptolina arctica* |  | 97.613 |
| KF422622 | *Rebecca salina* | *Chrysoculter rhomboideus* | 93.899 |
| L34669 | *Rebecca salina* |  | 94.960 |
| AF106059 | *Rebecca salina* |  | 94.695 |
| KU561125 | *Rebecca salina* |  | 94.960 |
| AF102987 | *Rebecca salina* |  | 93.634 |
| FR865774 | *Rebecca salina* |  | 93.634 |
| JF714244 | *Rebecca salina* |  | 92.838 |
| X90992 | *Reticulosphaera japonensis* |  | 97.613 |
| KF728656 | *Ruttnera lamellosa* | *Chrysotila lamellosa* | 97.082 |
| KF696664 | *Ruttnera lamellosa* | *Chrysotila lamellosa* | 97.082 |
| AM490998 | *Ruttnera lamellosa* |  | 97.082 |
| KT861320 | *Scyphosphaera apsteinii* |  | 97.878 |
| AM490984 | *Scyphosphaera apsteinii* |  | 97.878 |
| AY749614 | *Sphaerastrum fockii* |  | 81.698 |
| KF422621 | *Syracosphaera pulchra* |  | 97.878 |
| AM490987 | *Syracosphaera pulchra* |  | 97.878 |
| AB636317 | *Tergestiella adriatica* |  | 98.143 |
| AB636316 | *Tergestiella adriatica* |  | 98.143 |
| LC733216 | *Tisochrysis lutea* |  | 96.817 |
| AM490993 | *Umbilicosphaera hulburtiana* |  | 97.613 |
| AJ544118 | *Umbilicosphaera sibogae* |  | 97.878 |
| AJ544119 | *Umbilicosphaera sibogae var foliosa* |  | 97.613 |

| AB847979 | *Braarudosphaera bigelowii* |  | " |
| --- | --- | --- | --- |
| AB478413 | *Braarudosphaera bigelowii* |  | " |
| MH206612 | *Chrysochromulina parva* | 91.247 | " |
| MN727061 | *Chrysotila carterae* | 93.103 | " |
| MG022758 | *Chrysotila carterae* | 89.390 | " |
| MG022746 | *Chrysotila dentata* | 92.838 | " |
| MN186658 | *Chrysotila dentata* | 90.716 | " |
| OQ799064 | *Chrysotila stipitata* | 89.390 | " |
| OQ799063 | *Chrysotila stipitata* | 89.390 | " |
| OQ799062 | *Chrysotila stipitata* | 89.390 | " |
| U40924 | *Coccoid haptophyte* | 94.960 | " |
| KF899845 | *Dicrateria rotunda* |  | " |
| MG022751 | *Emiliania huxleyi* | 93.103 | " |
| KU600445 | *Isochrysis galbana* | 93.103 | " |
| KU600444 | *Isochrysis galbana* | 93.103 | " |
| KU600443 | *Isochrysis galbana* | 93.103 | " |
| KU600442 | *Isochrysis galbana* | 93.103 | " |
| KT852561 | *Isochrysis galbana* |  | unverified organism |
| OQ799059 | *Ochrosphaera neapolitana* | 89.920 | " |
| OQ799061 | *Ochrosphaera neapolitana* | 91.247 | " |
| OQ799060 | *Ochrosphaera neapolitana* | 92.308 | " |
| OQ799057 | *Ochrosphaera neapolitana* | 92.308 | " |
| PQ878102 | *Phaeocystis antarctica* | 93.899 | " |
| AJ278037 | *Phaeocystis globosa* |  | " |
| MN927485 | *Phaeocystis globosa* |  | " |
| AF166377 | Prymnesiophyte symbiont1 |  | symbiont |
| AF166376 | Prymnesiophyte symbiont3 |  | symbiont |
| AF166378 | Prymnesiophyte symbiont4 |  | symbiont |
| MN723534 | *Prymnesium parvum* | 95.225 | " |
| OQ799068 | *Ruttnera lamellosa* | 89.125 | " |
| OQ799067 | *Ruttnera lamelosa* | 90.981 | " |
| MN723153 | *Tisochrysis lutea* | 93.369 | " |
| AJ246275 | unclassified coccolithophorid |  | unclassified |

**(B)**: ITS2 sequences used and discarded

| **Genbank**  **accession** | **Species name**  **(obtained from literature)** | **Outdated species name**  **(obtained from NCBI)** | **Helix Transfer**  **(in %)** | **Template** |
| --- | --- | --- | --- | --- |
| AB180202 | *Chrysochromulina andersonii* |  | 67.78 | KT380974 |
| KT389974 | *Chrysochromulina simplex* |  | 98.89 | KT380974 |
| KT390082 | *Chrysochromulina simplex* |  | 68.89 | KT380974 |
| AM936919 | *Chrysotila elongata* | *Pleurochrysis elongata* | 67.59 | AM936920 |
| AM936920 | *Chrysotila pseudoroscoffensis* |  | 99.54 | AM936920 |
| AM936921 | *Chrysotila roscoffensis* |  | 99.54 | AM936920 |
| KC800939 | *Chrysotila roscoffensis* |  | 99.54 | AM936920 |
| AM936923 | *Chrysotila* sp*.* | *Pleurochrysis* sp. HAPPLH | 68.06 | AM936920 |
| AJ544122 | *Coccolithus braarudii* |  | 98.81 | AJ544122 |
| LC765394 | *Coccolithus braarudii* |  | 96.43 | AJ544122 |
| AJ544123 | *Coccolithus pelagicus* |  | 96.43 | AJ544122 |
| OR922675 | *Dicrateria rotunda* |  | 96.97 | OR922682 |
| OR922682 | *Dicrateria rotunda* |  | 98.99 | OR922682 |
| OR922701 | *Dicrateria rotunda* |  | 94.95 | OR922682 |
| OR922713 | *Dicrateria rotunda* |  | 94.95 | OR922682 |
| FJ946912 | *Isochrysis galbana* |  | 97.56 | FJ946913 |
| FJ946914 | *Isochrysis galbana* |  | 98.78 | FJ946913 |
| JX393297 | *Isochrysis galbana* |  | 98.78 | FJ946913 |
| JX393298 | *Isochrysis galbana* |  | 98.78 | FJ946913 |
| KC800941 | *Isochrysis galbana* |  | 98.78 | FJ946913 |
| KF998564 | *Isochrysis galbana* |  | 98.78 | FJ946913 |
| FJ946913 | *Isoschrysis galbana* |  | 98.78 | FJ946913 |
| AM690999 | *P. parvum f. parvum* | *Prymnesium parvum* | 98.85 | AM690999 |
| FJ907460 | *P. parvum f. parvum* | *Prymnesium parvum* | 97.10 | AM690999 |
| KJ756812 | *P. parvum f. parvum* | *Prymnesium parvum* | 96.55 | AM690999 |
| MK091108 | *P. parvum f. parvum* | *Prymnesium parvum* | 98.85 | AM690999 |
| MK091109 | *P. parvum f. parvum* | *Prymnesium parvum* | 98.85 | AM690999 |
| MK091110 | *P. parvum f. parvum* | *Prymnesium parvum* | 98.85 | AM690999 |
| MK091111 | *P. parvum f. parvum* | *Prymnesium parvum* | 98.85 | AM690999 |
| MK091112 | *P. parvum f. parvum* | *Prymnesium parvum* | 98.85 | AM690999 |
| MK091113 | *P. parvum f. parvum* | *Prymnesium parvum* | 98.85 | AM690999 |
| MK091114 | *P. parvum f. parvum* | *Prymnesium parvum* | 96.55 | AM690999 |
| MK091115 | *P. parvum f. parvum* | *Prymnesium parvum* | 96.55 | AM690999 |
| MK091116 | *P. parvum f. parvum* | *Prymnesium parvum* | 96.55 | AM690999 |
| MK091117 | *P. parvum f. parvum* | *Prymnesium parvum* | 96.55 | AM690999 |
| MK091118 | *P. parvum f. parvum* | *Prymnesium parvum* | 96.55 | AM690999 |
| MK091119 | *P. parvum f. parvum* | *Prymnesium parvum* | 96.55 | AM690999 |
| MK091120 | *P. parvum f. parvum* | *Prymnesium parvum* | 96.55 | AM690999 |
| MK091121 | *P. parvum f. parvum* | *Prymnesium parvum* | 96.55 | AM690999 |
| MK091122 | *P. parvum f. parvum* | *Prymnesium parvum* | 97.70 | AM690999 |
| MK091123 | *P. parvum f. parvum* | *Prymnesium parvum* | 95.40 | AM690999 |
| MK091124 | *P. parvum f. parvum* | *Prymnesium parvum* | 95.40 | AM690999 |
| MK091125 | *P. parvum f. parvum* | *Prymnesium parvum* | 95.40 | AM690999 |
| MK091126 | *P. parvum f. parvum* | *Prymnesium parvum* | 95.40 | AM690999 |
| MK091127 | *P. parvum f. parvum* | *Prymnesium parvum* | 95.40 | AM690999 |
| MK091128 | *P. parvum f. parvum* | *Prymnesium parvum* | 95.40 | AM690999 |
| MK091129 | *P. parvum f. parvum* | *Prymnesium parvum* | 95.40 | AM690999 |
| MK091130 | *P. parvum f. parvum* | *Prymnesium parvum* | 95.40 | AM690999 |
| MK091131 | *P. parvum f. parvum* | *Prymnesium parvum* | 95.40 | AM690999 |
| MK091132 | *P. parvum f. parvum* | *Prymnesium parvum* | 91.95 | AM690999 |
| MK091133 | *P. parvum f. parvum* | *Prymnesium parvum* | 91.95 | AM690999 |
| MN727031 | *P. parvum f. parvum* | *Prymnesium parvum* | 96.55 | AM690999 |
| MW471019 | *P. parvum f. parvum* | *Prymnesium parvum* | 96.55 | AM690999 |
| MZ823500 | *P. parvum f. parvum* | *Prymnesium parvum* | 95.40 | AM690999 |
| PP660306 | *P. parvum f. parvum* | *Prymnesium parvum* | 98.85 | AM690999 |
| PP660307 | *P. parvum f. parvum* | *Prymnesium parvum* | 98.85 | AM690999 |
| PP660308 | *P. parvum f. parvum* | *Prymnesium parvum* | 98.85 | AM690999 |
| PP660309 | *P. parvum f. parvum* | *Prymnesium parvum* | 98.85 | AM690999 |
| PQ288669 | *P. parvum f. parvum* | *Prymnesium parvum* | 95.40 | AM690999 |
| MN603176 | *Phaeocysis globosa* |  | 99.24 | AJ279504 |
| AJ279500 | *Phaeocystis globosa* |  | 90.84 | AJ279504 |
| AJ279501 | *Phaeocystis globosa* |  | 96.95 | AJ279504 |
| AJ279502 | *Phaeocystis globosa* |  | 96.18 | AJ279504 |
| AJ279503 | *Phaeocystis globosa* |  | 96.18 | AJ279504 |
| AJ279504 | *Phaeocystis globosa* |  | 96.18 | AJ279504 |
| AJ279505 | *Phaeocystis globosa* |  | 99.24 | AJ279504 |
| EU024766 | *Phaeocystis globosa* |  | 96.95 | AJ279504 |
| EU077557 | *Phaeocystis globosa* |  | 96.95 | AJ279504 |
| KT390022 | *Phaeocystis globosa* |  | 98.47 | AJ279504 |
| KT390053 | *Phaeocystis globosa* |  | 98.47 | AJ279504 |
| MN603166 | *Phaeocystis globosa* |  | 99.24 | AJ279504 |
| MN603167 | *Phaeocystis globosa* |  | 98.47 | AJ279504 |
| MN603168 | *Phaeocystis globosa* |  | 99.24 | AJ279504 |
| MN603269 | *Phaeocystis globosa* |  | 97.71 | AJ279504 |
| MN603170 | *Phaeocystis globosa* |  | 97.71 | AJ279504 |
| MN603171 | *Phaeocystis globosa* |  | 96.95 | AJ279504 |
| MN603172 | *Phaeocystis globosa* |  | 99.24 | AJ279504 |
| MN603173 | *Phaeocystis globosa* |  | 96.95 | AJ279504 |
| MN603174 | *Phaeocystis globosa* |  | 99.24 | AJ279504 |
| MN603175 | *Phaeocystis globosa* |  | 96.95 | AJ279504 |
| MN603177 | *Phaeocystis globosa* |  | 98.47 | AJ279504 |
| MN603178 | *Phaeocystis globosa* |  | 98.47 | AJ279504 |
| MN603179 | *Phaeocystis globosa* |  | 96.95 | AJ279504 |
| MN603180 | *Phaeocystis globosa* |  | 96.95 | AJ279504 |
| MN603181 | *Phaeocystis globosa* |  | 98.47 | AJ279504 |
| MN603182 | *Phaeocystis globosa* |  | 98.47 | AJ279504 |
| MN603183 | *Phaeocystis globosa* |  | 99.24 | AJ279504 |
| MN603184 | *Phaeocystis globosa* |  | 96.95 | AJ279504 |
| MN603185 | *Phaeocystis globosa* |  | 98.47 | AJ279504 |
| MN603186 | *Phaeocystis globosa* |  | 99.24 | AJ279504 |
| MN603187 | *Phaeocystis globosa* |  | 99.24 | AJ279504 |
| MN603188 | *Phaeocystis globosa* |  | 99.24 | AJ279504 |
| MN603189 | *Phaeocystis globosa* |  | 97.71 | AJ279504 |
| MN603190 | *Phaeocystis globosa* |  | 99.24 | AJ279504 |
| MN603191 | *Phaeocystis globosa* |  | 99.24 | AJ279504 |
| MN603192 | *Phaeocystis globosa* |  | 97.71 | AJ279504 |
| MN603193 | *Phaeocystis globosa* |  | 97.71 | AJ279504 |
| MN603194 | *Phaeocystis globosa* |  | 98.47 | AJ279504 |
| MN603195 | *Phaeocystis globosa* |  | 99.24 | AJ279504 |
| MN603196 | *Phaeocystis globosa* |  | 98.47 | AJ279504 |
| MN603197 | *Phaeocystis globosa* |  | 98.47 | AJ279504 |
| MN603198 | *Phaeocystis globosa* |  | 98.47 | AJ279504 |
| MN603199 | *Phaeocystis globosa* |  | 99.24 | AJ279504 |
| MN603200 | *Phaeocystis globosa* |  | 98.47 | AJ279504 |
| MN603201 | *Phaeocystis globosa* |  | 97.71 | AJ279504 |
| MN603202 | *Phaeocystis globosa* |  | 97.71 | AJ279504 |
| MN603203 | *Phaeocystis globosa* |  | 98.47 | AJ279504 |
| MN603204 | *Phaeocystis globosa* |  | 99.24 | AJ279504 |
| MN603205 | *Phaeocystis globosa* |  | 98.47 | AJ279504 |
| MN603206 | *Phaeocystis globosa* |  | 99.24 | AJ279504 |
| MN603207 | *Phaeocystis globosa* |  | 97.71 | AJ279504 |
| MN603208 | *Phaeocystis globosa* |  | 98.47 | AJ279504 |
| MN603209 | *Phaeocystis globosa* |  | 99.24 | AJ279504 |
| MN603210 | *Phaeocystis globosa* |  | 99.24 | AJ279504 |
| MN603211 | *Phaeocystis globosa* |  | 98.47 | AJ279504 |
| MN603212 | *Phaeocystis globosa* |  | 98.47 | AJ279504 |
| MN603213 | *Phaeocystis globosa* |  | 98.47 | AJ279504 |
| MN603214 | *Phaeocystis globosa* |  | 99.24 | AJ279504 |
| MN603215 | *Phaeocystis globosa* |  | 99.24 | AJ279504 |
| MN603216 | *Phaeocystis globosa* |  | 99.24 | AJ279504 |
| MN603217 | *Phaeocystis globosa* |  | 98.47 | AJ279504 |
| MN603218 | *Phaeocystis globosa* |  | 99.24 | AJ279504 |
| MN603219 | *Phaeocystis globosa* |  | 98.47 | AJ279504 |
| MN603220 | *Phaeocystis globosa* |  | 99.24 | AJ279504 |
| MN603221 | *Phaeocystis globosa* |  | 98.47 | AJ279504 |
| MN603222 | *Phaeocystis globosa* |  | 99.24 | AJ279504 |
| OP686589 | *Phaeocystis globosa* |  | 96.95 | AJ279504 |
| PP828742 | *Phaeocystis globosa* |  | 96.95 | AJ279504 |
| AJ271046 | *Phaeocystis pouchetii* |  | 75.57 | AJ279504 |
| AJ417693 | *Phaeocystis pouchetii* |  | 71.76 | AJ279504 |
| KT390023 | *Phaeocystis* sp. JD_2012 |  | 97.71 | AJ279504 |
| AF289038 | *Prymnesium parvum f. patelliferum* |  | 98.85 | AM690999 |
| MW471018 | *Prymnesium parvum f. patelliferum* |  | 95.40 | AM690999 |
| MZ376741 | *Prymnesium* sp. |  | 94.25 | AM690999 |
| KC817139 | *Prymnesium* sp. IOAC727S |  | 95.40 | AM690999 |
| MZ376740 | *Prymnesium* sp. IOAC727S |  | 95.40 | AM690999 |
| FJ946911 | *Tisochrysis lutea* | *Isochrysis* sp. CCAP_927_14 | 98.78 | FJ946913 |

| LC819336 | *Chrysochromulina simplex* |  | “ |  |
| --- | --- | --- | --- | --- |
| KT389902 | *Chrysochromulina simplex* | 54.44 | / | KT380974 |
| LC819335 | *Chrysochromulina simplex* | 56.67 | / | KT380974 |
| KT389978 | *Chrysochromulina simplex* | 62.22 | / | KT380974 |
| AM936918 | *Chrysotila carterae* |  | “ |  |
| PP828743 | *Chrysotila dentata* | 50.46 | / | AM936920 |
| MW471017 | *Chrysotila pseudoroscoffensis* |  | “ |  |
| AM936922 | *Chrysotila scherfelli* |  | “ |  |
| MN791087 | *Diacronema lutheri* | 37.93 | / | AM690999 |
| FJ946909 | *Isochrysis galbana* | 56.1 | / | FJ946913 |
| FJ946910 | *Isochrysis galbana* | 56.1 | / | FJ946913 |
| MH298754 | *Isochrysis nuda* | 57.32 | / | FJ946913 |
| PP818641 | *Isochrysis* sp. | 58.54 | / | FJ946913 |
| FR865766 | *Monochrysis* sp. CCAP_931_2 | 43.68 | / | AM690999 |
| FR865771 | *Pavlova gyrans* | 35.71 | / | AJ544122 |
| FR865772 | *Pavlova gyrans* | 41.67 | / | AJ544122 |
| FR865773 | *Pavlova* sp. CCAP_940_2 | 35.56 | / | KT380974 |
| AF289040 | *Phaeocystis antarctica* |  | “ |  |
| AF289039 | *Phaeocystis antarctica* |  | “ |  |
| KT389890 | *Phaeocystis antarctica* | 50.0 | / | FJ946913 |
| KT389906 | *Phaeocystis antarctica* | 45.12 | / | FJ946913 |
| KT389884 | *Phaeocystis antarctica* | 60.71 | / | AJ544122 |
| KT390140 | *Phaeocystis antarctica* | 63.1 | / | AJ544122 |
| PV016894 | *Phaeocystis globosa* |  | “ |  |
| PP828741 | *Phaeocystis globosa* |  | “ |  |
| MN603086 | *Phaeocystis globosa* |  | “ |  |
| MN603085 | *Phaeocystis globosa* |  | “ |  |
| MN603084 | *Phaeocystis globosa* |  | “ |  |
| MN603083 | *Phaeocystis globosa* |  | “ |  |
| MN603082 | *Phaeocystis globosa* |  | “ |  |
| MN603081 | *Phaeocystis globosa* |  | “ |  |
| MN603080 | *Phaeocystis globosa* |  | “ |  |
| MN603079 | *Phaeocystis globosa* |  | “ |  |
| MN603078 | *Phaeocystis globosa* |  | “ |  |
| MN603077 | *Phaeocystis globosa* |  | “ |  |
| MN603076 | *Phaeocystis globosa* |  | “ |  |
| MN603075 | *Phaeocystis globosa* |  | “ |  |
| MN603074 | *Phaeocystis globosa* |  | “ |  |
| MN603073 | *Phaeocystis globosa* |  | “ |  |
| MN603072 | *Phaeocystis globosa* |  | “ |  |
| MN603071 | *Phaeocystis globosa* |  | “ |  |
| MN603070 | *Phaeocystis globosa* |  | “ |  |
| MN603069 | *Phaeocystis globosa* |  | “ |  |
| MN603068 | *Phaeocystis globosa* |  | “ |  |
| MN603067 | *Phaeocystis globosa* |  | “ |  |
| MN603066 | *Phaeocystis globosa* |  | “ |  |
| MN603065 | *Phaeocystis globosa* |  | “ |  |
| MN603064 | *Phaeocystis globosa* |  | “ |  |
| MN603063 | *Phaeocystis globosa* |  | “ |  |
| MN603062 | *Phaeocystis globosa* |  | “ |  |
| MN603061 | *Phaeocystis globosa* |  | “ |  |
| MN603060 | *Phaeocystis globosa* |  | “ |  |
| MN603059 | *Phaeocystis globosa* |  | “ |  |
| MN603058 | *Phaeocystis globosa* |  | “ |  |
| MN603057 | *Phaeocystis globosa* |  | “ |  |
| MN603009 | *Phaeocystis globosa* |  | “ |  |
| MN603008 | *Phaeocystis globosa* |  | “ |  |
| MN603007 | *Phaeocystis globosa* |  | “ |  |
| GQ118978 | *Phaeocystis globosa* |  | “ |  |
| GQ118977 | *Phaeocystis globosa* |  | “ |  |
| GQ118976 | *Phaeocystis globosa* |  | “ |  |
| GQ118975 | *Phaeocystis globosa* |  | “ |  |
| GQ118974 | *Phaeocystis globosa* |  | “ |  |
| GQ118973 | *Phaeocystis globosa* |  | “ |  |
| GQ118972 | *Phaeocystis globosa* |  | “ |  |
| AJ271217 | *Phaeocystis* sp. |  | “ |  |
| AJ271218 | *Phaeocystis* sp*.* |  | “ |  |
| KY095035 | *Phaeocystis* sp. |  | “ |  |
| KY095039 | *Phaeocystis* sp*.* |  | “ |  |
| KT389873 | *Phaeocystis* sp. JD_2012 | 54.44 | / | KT380974 |
| PP426008 | *Prymnesium parvum* |  | “ |  |
| PP426009 | *Prymnesium parvum* |  | “ |  |
| PP42010 | *Prymnesium parvum* |  | “ |  |
| PP426011 | *Prymnesium parvum* |  | “ |  |
| PP426012 | *Prymnesium parvum* |  | “ |  |
| PP426013 | *Prymnesium parvum* |  | “ |  |
| PP426014 | *Prymnesium parvum* |  | “ |  |
| OR231183 | *Prymnesium parvum* |  | “ |  |
| PV016891 | *Prymnesium parvum* f. *patelliferum* |  | “ |  |
| KT389864 | *Prymnesium parvum* f. *patelliferum* | 55.17 | / | AM690999 |
| KT389875 | *Prymnesium parvum* f. *patelliferum* | 55.17 | / | AM690999 |
| KT389870 | *Prymnesium parvum* f. *patelliferum* | 63.33 | / | KT380974 |
| KT389881 | *Prymnesium parvum* f. *patelliferum* | 63.33 | / | KT380974 |
| FR865770 | *Prymnesium pigrum* | 50.58 | / | AM690999 |
| FR865774 | *Rebecca salina* | 29.27 | / | FJ946913 |
| KM365434 | *Tisochrysis lutea* |  | “ |  |
