# Supplementary material for: ITS2 and 18S rRNA gene sequence-structure phylogeny of the Haptophyta (Haptista)
Source: PLoS One. 2026 Mar 19;21(3):e0344353. doi: 10.1371/journal.pone.0344353 (PMC13001949; doi:10.1371/journal.pone.0344353)
Supplement: S2 Table — (A) Table showing the most interesting results of comparing all 18S sequence-only and sequence-structure trees to each other. (B) Table showing the most interesting results of comparing all ITS2 sequence-only and sequence-structure trees to each other. (DOCX) [file pone.0344353.s002.docx]

**(A) 18S table summarizing important findings for different methods**

| 18S | NJ-overview | PNJ-original | ML-subset |
| --- | --- | --- | --- |
| Sequence-structure | -6 monophyletic orders  -monophyletic Calcidiscaceae (including Hayaster) | -6 monophyletic orders  -monophyletic Calcidiscaceae (including Hayaster)  -backbone support higher compared to subset trees | -7 monophyletic orders  -monophyletic Calcidiscaceae (including Hayaster)  -monophyletic Prymnesiales  -monophyletic Prymnesiaceae  -closest to literature |
| Sequence-only | -6 monophyletic orders | -6 monophyletic orders  - monophyletic Prymnesiales  -backbone support higher compared to subset trees | -6 monophyletic orders  -monophyletic Prymnesiaceae |

**(B) ITS2 table summarizing important findings for different methods**

| **ITS2** | **NJ-overview** | **PNJ-original** | **ML-subset** |
| --- | --- | --- | --- |
| **Sequence-structure** | -3 monophyletic orders  -monophyletic Prymnesiales | -3 monophyletic orders  -backbone better supported than in Sequence-only  -monophyletic Prymnesiales | -2 monophyletic orders  -backbone better supported than in Sequence-only |
| **Sequence-only** | -2 monophyletic orders  -*Chrysochromulina* were not monophyletic  -topology strongly deviating from literature | -2 monophyletic orders  -*Chrysochromulina* were not monophyletic  -topology strongly deviating from literature  -no support for subclades defined as profiles | -2 monophyletic orders  -Prymnesiaceae were not monophyletic  -topology strongly deviating from literature |
