# Supplementary figures and images for: ITS2 and 18S rRNA gene sequence-structure phylogeny of the Haptophyta (Haptista)
Source: PLoS One. 2026 Mar 19;21(3):e0344353. doi: 10.1371/journal.pone.0344353 (PMC13001949; doi:10.1371/journal.pone.0344353)

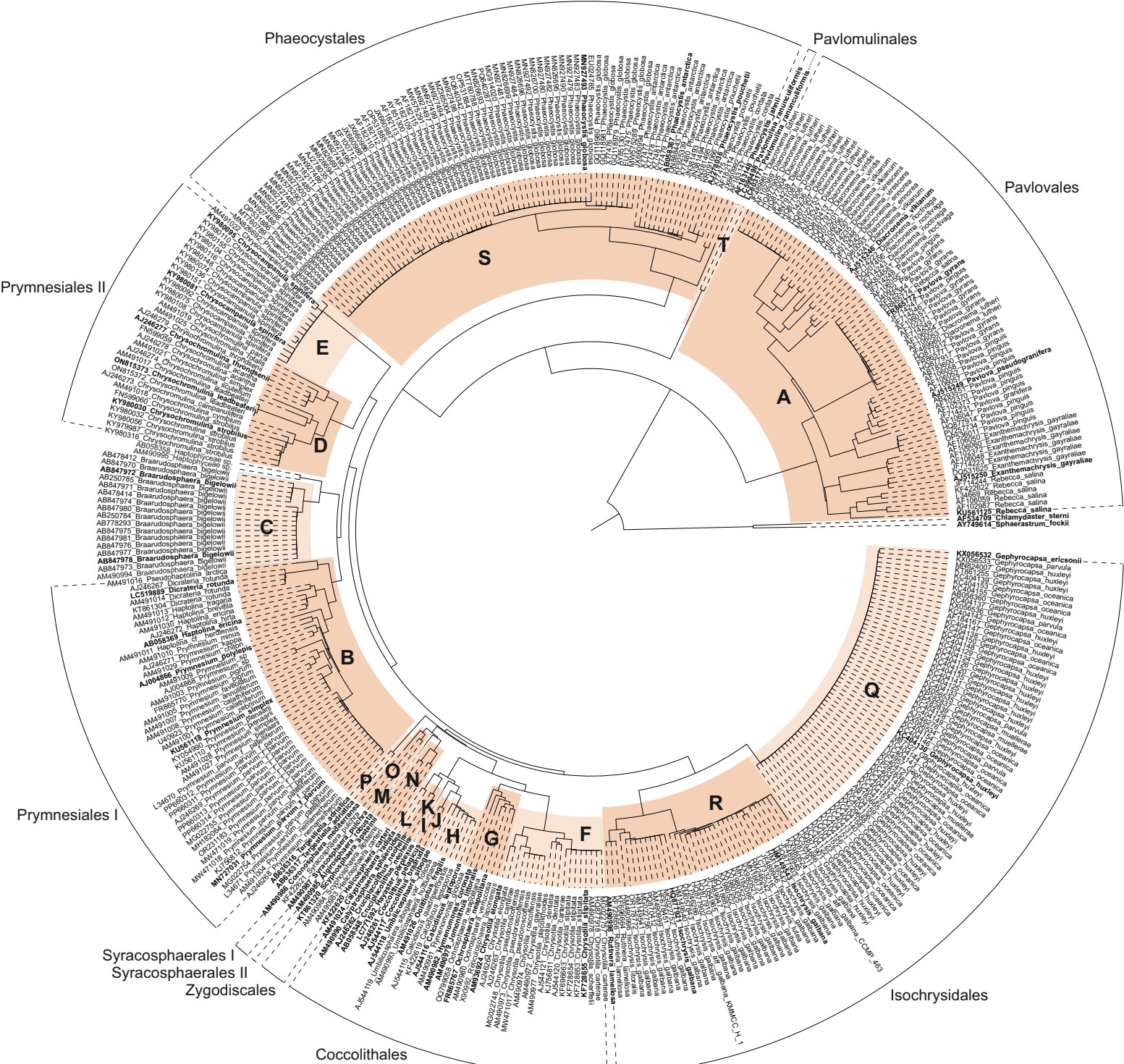

Supplement: S1 Fig — The sequence-only alignment was generated using ClustalX. Species names are accompanied by their respective GenBank accession numbers. According to (Edvardsen et al. 2011, Piwosz et al. 2019, Larsen 1999, Archontikis et al. 2023, Andersen et al. 2014, Bendif et al. 2013, Andersen et al. 2014) some species are renamed in contrast to NCBI taxonomy (cf. S1 Table). Clades selected for subsequent profile Neighbor-Joining (PNJ) analyses are alternately colored, and sequences used in later subset analyses are highlighted in bold. Families and orders within the haptophytes are labeled A–T as follows: A: Pavlovaceae, B: Prymnesiaceae I, C: Braarudosphaeraceae, D: Chrysochromulinaceae, E: Prymnesiaceae II, F: Chrysotilaceae, G: Hymenomonadaceae, H: Calcidiscaceae I, I: Hayaster perplexus (Calcidiscaceae II), J: Coccolithaceae I, K: Coccolithaceae II, L: Calyptrosphaeraceae, M: Rhabdosphaeraceae, N: Zygodiscales (including Pontosphaeraceae & Helicosphaeraceae), O: Syracosphaeraceae, P: Tergestiella adriatica (Watznaueriaceae), Q: Noelaerhabdaceae, R: Isochrysidaceae, S: Phaeocystaceae, T: Pavlomulinaceae. Orders are additionally annotated around the tree. The scale bar and branch lengths represent evolutionary distances. (PDF) [file pone.0344353.s003.pdf]

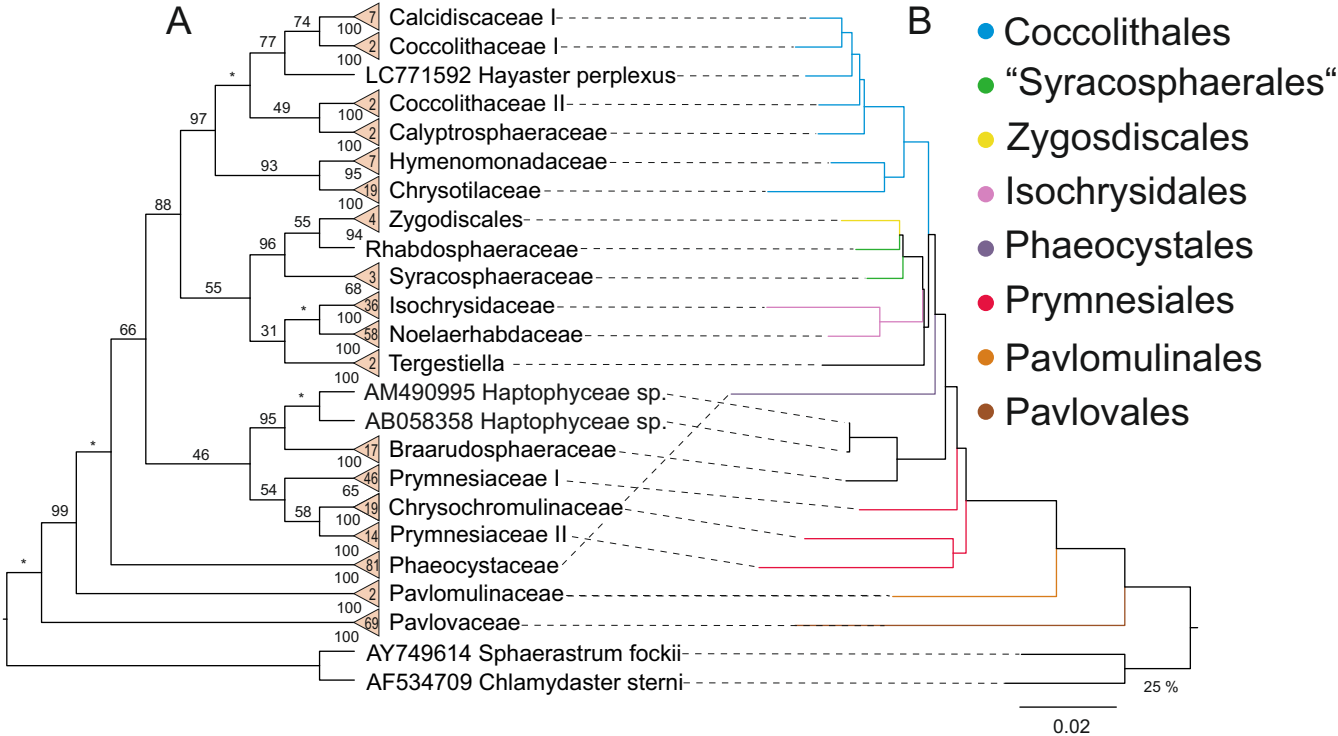

Supplement: S2 Fig — The tree was generated using ProfDistS, and the sequence-only alignment was generated using ClustalX. Species names are accompanied by their respective GenBank accession numbers. Two strains (GenBank accession AM490995, AB058358) were wrongly classified in NCBI (Edvardsen et al. 2011, Piwosz et al. 2019) and therefore renamed to Haptophyceae sp. (for outdated names see S1 Table). A: The cladogram on the left shows a three-times iterated PNJ tree. Bootstrap values from 100 pseudo-replicates are displayed at internal nodes. During each iteration, super-profiles were generated using existing profiles and bootstrap values >75. Numbers within the triangles indicate the number of sequences in manually defined profiles. Profile bootstrap values were derived and transferred from bootstrap testing (100 pseudo-replicates) performed on the overview NJ tree (S1 Fig). An asterisk (*) stands for a bootstrap value of 100. B: The phylogram on the right depicts the original PNJ tree without further iterations. Both trees are rooted with Chlamydaster sterni (GenBank accession AF534709) and Sphaerastrum fockii (GenBank accession AY749614). Branch lengths and the scale bar represent evolutionary distances. Colours indicate the corresponding haptophyte orders as follows: blue: Coccolithales, green: Syracosphaerales, yellow: Zygodiscales, pink: Isochrysidales, violet: Phaeocystales, red: Prymnesiales, orange: Pavlomulinales, brown: Pavlovales. (PDF) [file pone.0344353.s004.pdf]

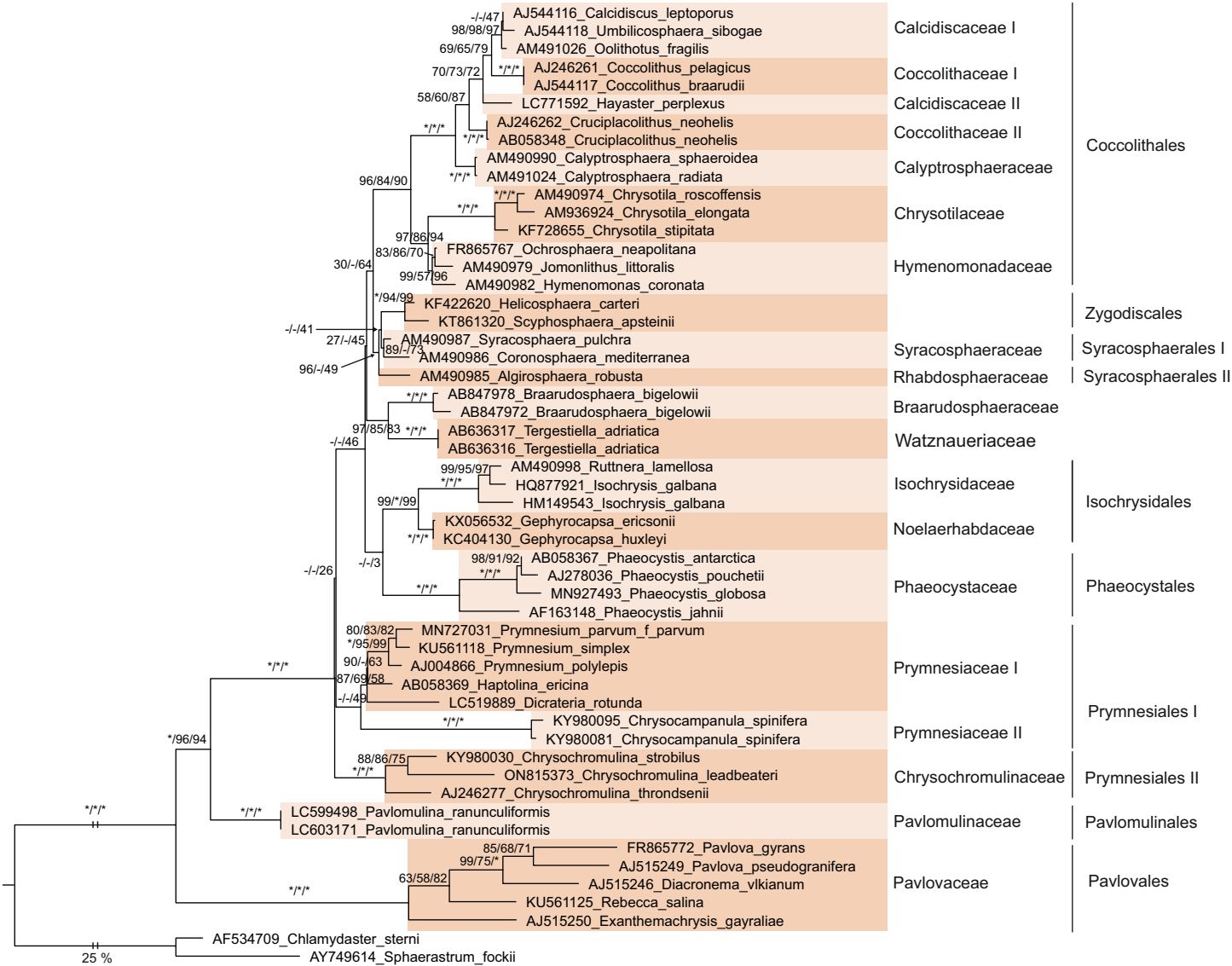

Supplement: S3 Fig — The tree was constructed in MEGA using the TN93 + G + I model. The sequence-only alignment was generated using ClustalX. Some species (cf. S1 Table) were named differently than in NCBI according to (Larsen 1999, Archontikis et al. 2023, Bendif et al. 2013, Andersen et al. 2014). Alternating colors highlight the PNJ profiles, and family and order names are displayed on the right side of the tree. Non-monophyletic orders are indicated by Roman numerals. The branch length of the outgroup (Sphaerastrum fockii and Chlamydaster sterni) is trimmed to 25%. Branch lengths and the scale bar represent evolutionary distances. Bootstrap values are shown for three methods—neighbor-joining (NJ, obtained in ProfDistS), maximum-parsimony (MP, obtained in PAUP), and maximum-likelihood (ML)—in the order NJ/MP/ML. A bootstrap value of 100 is marked with an asterisk (*), while a dash (-) indicates differing tree topologies across methods. (PDF) [file pone.0344353.s005.pdf]

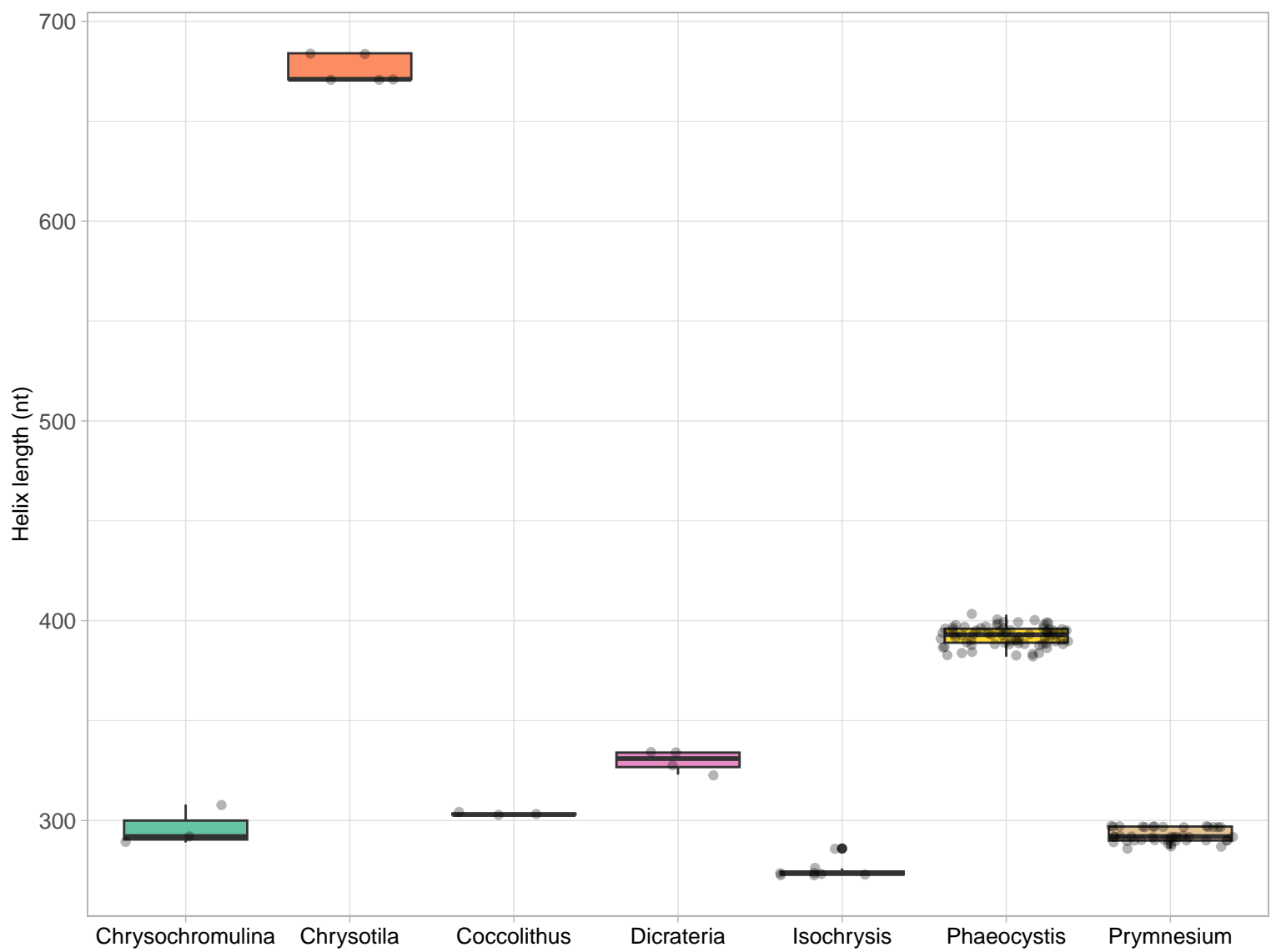

Supplement: S4 Fig — The sequence lengths were obtained from all sequences used in the ITS2 analysis. The length distribution is shown for Chrysochromulina (turquois), Chrysotila (orange), Coccolithus (blue), Dicrateria (violet), Isochrysis (lightgreen), Phaeocystis (yellow) and Prymnesium (beige). (PDF) [file pone.0344353.s006.pdf]

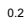

Supplement: S5 Fig — The sequence-only alignment was generated using ClustalX. Species names are accompanied by their respective GenBank accession numbers. According to (Larsen 1999, Andersen et al. 2014, Bendif et al. 2013) species are renamed in contrast to NCBI taxonomy (cf. S1 Table). As the outgroup, Phaeocystales was chosen. Clades selected for subsequent profile Neighbor-Joining (PNJ) analyses are alternately colored, and sequences used in later subset analyses are highlighted in bold. Families and orders within Prymnesiophyceae are labeled A–H as follows: A: Phaeocystis, B: Prymnesium, C: Dicrateria, D: Coccolithus, E: Chrysotila, F: Chrysochromulina I, G: Isochrysis, H: Chrysochromulina andersonii. Orders are additionally annotated around the tree. The scale bar and branch lengths represent evolutionary distances. (PDF) [file pone.0344353.s007.pdf]

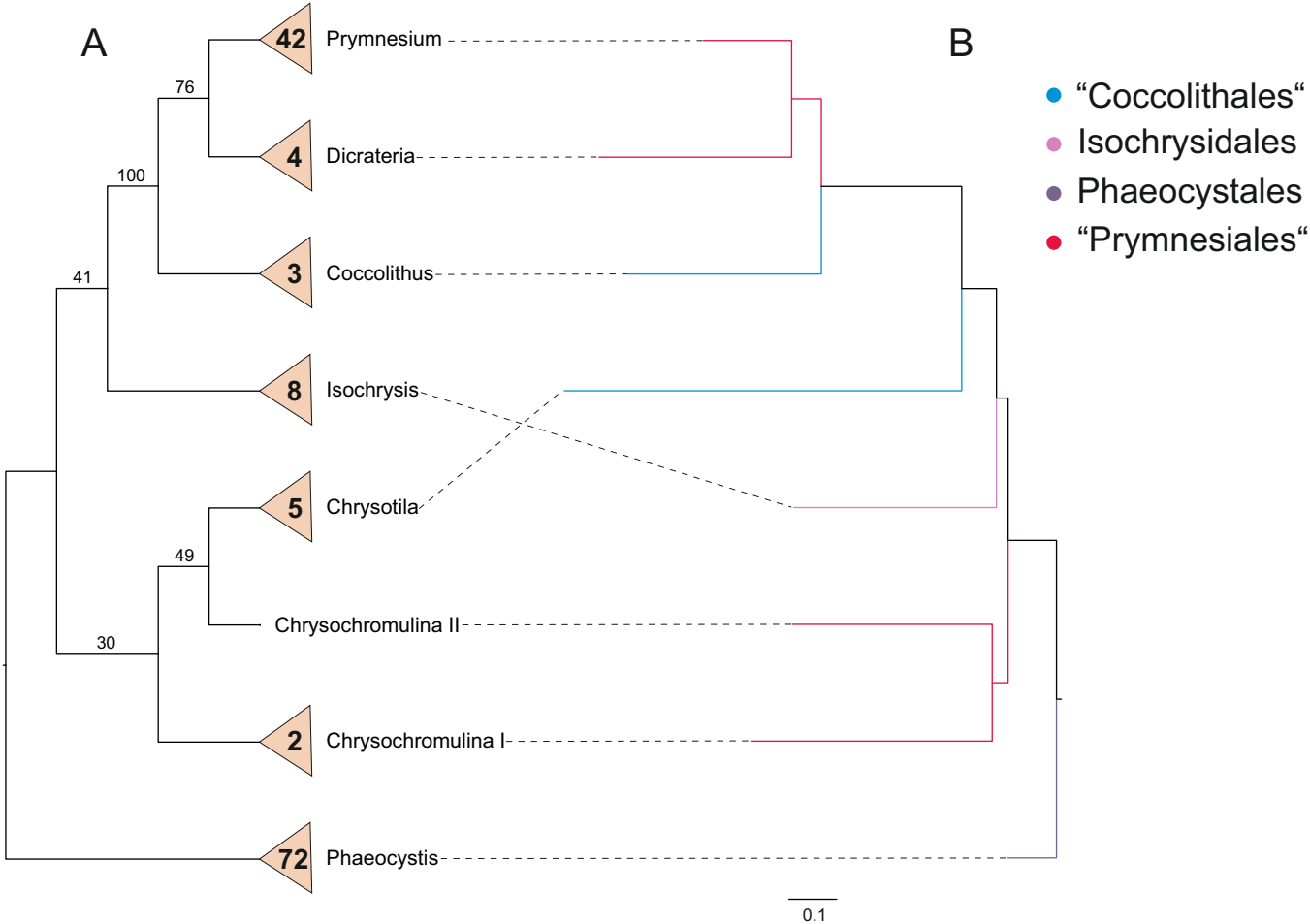

Supplement: S6 Fig — The tree was generated using ProfDistS, and the sequence-only alignment was generated using ClustalX. A: The cladogram on the left shows a three-times iterated PNJ tree. Bootstrap values from 100 pseudo-replicates are displayed at internal nodes. During each iteration, super-profiles were generated using existing profiles and bootstrap values >75. Numbers within the triangles indicate the number of sequences in manually defined profiles. Profile bootstrap values could not be obtained due to polytomy. B: The phylogram on the right depicts the original PNJ tree without further iterations. Both trees are rooted with Phaeocystales. Branch lengths and the scale bar represent evolutionary distances. Colours indicate the corresponding haptophyte orders as follows: blue: Coccolithales, pink: Isochrysidales, violet: Phaeocystales, red: Prymnesiales. (PDF) [file pone.0344353.s008.pdf]

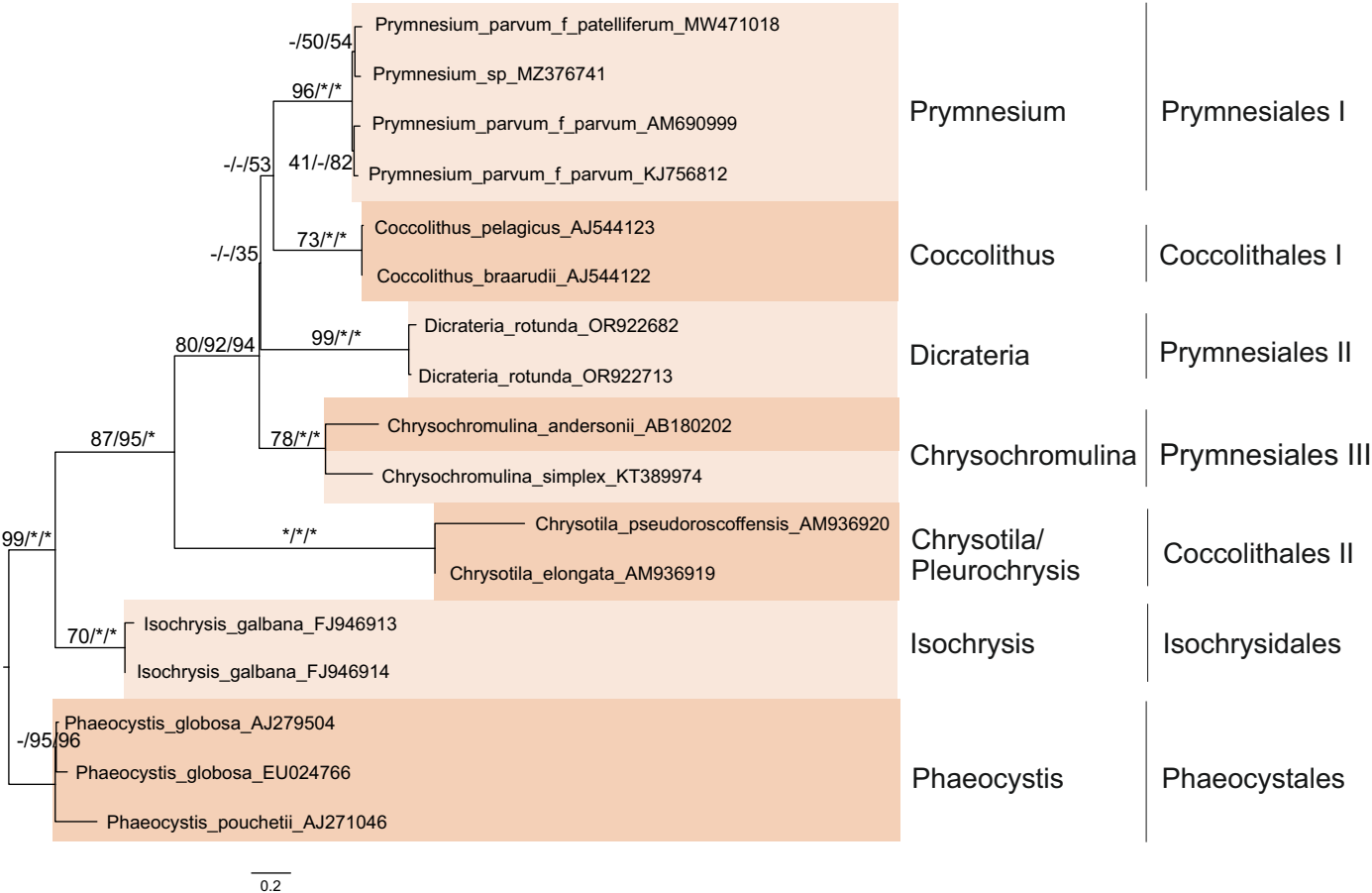

Supplement: S7 Fig — The tree was constructed in MEGA using the HKY + G model. The sequence-only alignment was generated using ClustalX. According to (Andersen et al. 2014, Larsen 1999) species are renamed in contrast to NCBI taxonomy (cf. S1 Table). Alternating colors highlight the PNJ profiles. Genera and order names are displayed on the right side of the tree. Non-monophyletic orders are indicated by Roman numerals. Phaeocystales was chosen as outgroup. Branch lengths and the scale bar represent evolutionary distances. Bootstrap values are shown for three methods—neighbor-joining (NJ, obtained in MEGA using the Maximum-Composite-Likelihood+G model as replacement for HKY + G), maximum-parsimony (MP, obtained in PAUP), and maximum-likelihood (ML)—in the order NJ/MP/ML. A bootstrap value of 100 is marked with an asterisk (*), while a dash (-) indicates differing tree topologies across methods. (PDF) [file pone.0344353.s009.pdf]
